# Interaction Between Age and Individual Heterogeneity Shapes Breeding Probability in a Long‐Lived Marine Ectotherm

**DOI:** 10.1002/ece3.72430

**Published:** 2025-11-09

**Authors:** C. George Glen, Jean‐Dominique Lebreton, Walter Mustin, Karen A. Bjorndal

**Affiliations:** ^1^ Archie Carr Center for Sea Turtle Research University of Florida Gainesville Florida USA; ^2^ Department of Biology University of Florida Gainesville Florida USA; ^3^ Centre D'ecologie Fonctionnelle et Evolutive, UMR 5175 CNRS Université de Montpellier Montpellier France; ^4^ Cayman Turtle Center Grand Cayman, West Bay Cayman Islands

**Keywords:** breeding probability, individual heterogeneity, sea turtles, selective disappearance, senescence

## Abstract

In iteroparous species, reproductive skipping is generally considered an adaptive strategy. Non‐breeding individuals should have a greater annual survival probability and retain greater future reproductive potential. Yet, the role of age on changes in breeding probability remains untested in many long‐lived testudines. To bridge this knowledge gap, we leveraged a 52‐year dataset on captive green turtles, an ancient lineage of marine ectotherms. Sea turtles serve as an interesting model system because they exhibit a reproductive strategy characterized by delayed maturity followed by intense reproductive bursts. Using a multi‐event capture‐mark‐recapture framework, our results reveal that individual quality and age were the primary drivers of reproductive patterns. High‐quality turtles were more likely to remain breeders in consecutive years, and low‐quality turtles were more likely to remain non‐breeders, an effect that became more dramatic at older ages. Furthermore, there was an antagonistic relationship between age and breeding experience on the waiting time between breeding seasons. At the population level, we found evidence of actuarial [survival] senescence but negligible reproductive senescence, with females maintaining a high residual reproductive value into old age. Collectively, our findings demonstrate the role of lifelong individual differences in shaping life histories, a fact that has been historically overlooked in long‐lived marine vertebrates like sea turtles, largely due to the immense logistical challenge of monitoring individuals over timespans that may equal a single academic career.

## Graphical abstract

Using a 52‐year dataset on captive green turtles, our study reveals that individual quality and age are the primary drivers of reproductive patterns. We found that high‐quality turtles bred more consistently throughout their lives and that females maintained a high reproductive value into old age despite evidence of physical aging. These findings demonstrate the critical and historically overlooked role of lifelong individual differences in shaping the life histories of long‐lived marine vertebrates.

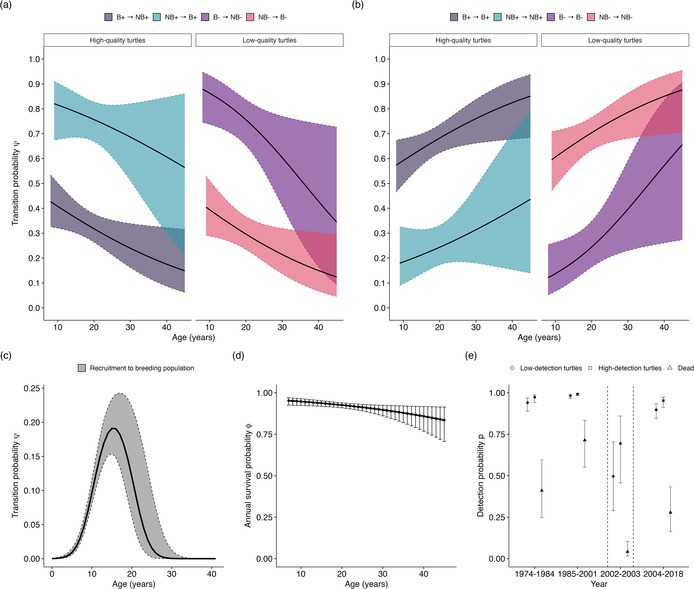

## 1 Introduction

A central premise in life history theory is that reproducing is expensive (Clutton‐Brock 1988; Stearns 1992). Reproductive schedules, which govern the timing and frequency of breeding events over a lifespan, are shaped by fundamental trade‐offs that balance the costs of current reproduction against investment in future survival (Charnov and Krebs 1974; Stearns 1992; Williams 1966). In long‐lived iteroparous species, restraining reproductive investment under unfavorable conditions is an adaptive strategy (Bull and Shine 1979). When reproduction in poor years carries a larger survival risk to the parent or offspring, reproductive skipping can increase an organism's expected contribution to future generations (Cam et al. 1998; Clutton‐Brock 1988). Despite a strong theoretical foundation, the effect of age and reproductive effort on longevity and breeding schedules remains poorly understood in many long‐lived taxa.

At a proximate level, the decision to reproduce is contingent on endogenous reserves surpassing a critical threshold (Erikstad et al. 1998; McNamara and Houston 1996). As a result, reproductive skipping is expected to be more common in temporally varying environments (Orzack and Tuljapurkar 2001; Reed et al. 2015; Skjæraasen et al. 2012). However, breeding thresholds and reproductive costs are unlikely to be fixed and often exhibit a plastic response to environmental conditions that occur synergistically with or independent from physiological factors such as age (Cooper and Kaplan 1982). Age‐related shifts in reproductive costs, driven by physiological changes, directly influence demographic rates like breeding probability (Beauplet et al. 2006). For instance, a greater investment in reproduction early in life may result in an earlier onset and a more intense rate of actuarial senescence (Hayward et al. 2015). Although, the removal of animals from a breeding population may also reflect a decreased breeding probability that may occur independent from changes in fecundity. This makes senescence—characterized by age‐related declines in survival and reproduction—a critical but multifaceted factor in understanding the evolution of life histories. To first link proximate drivers of reproductive decisions with evolutionary forces governing the expression of senescence, we must grapple with a fundamental question: why do organisms senesce at all?

**FIGURE 1:**
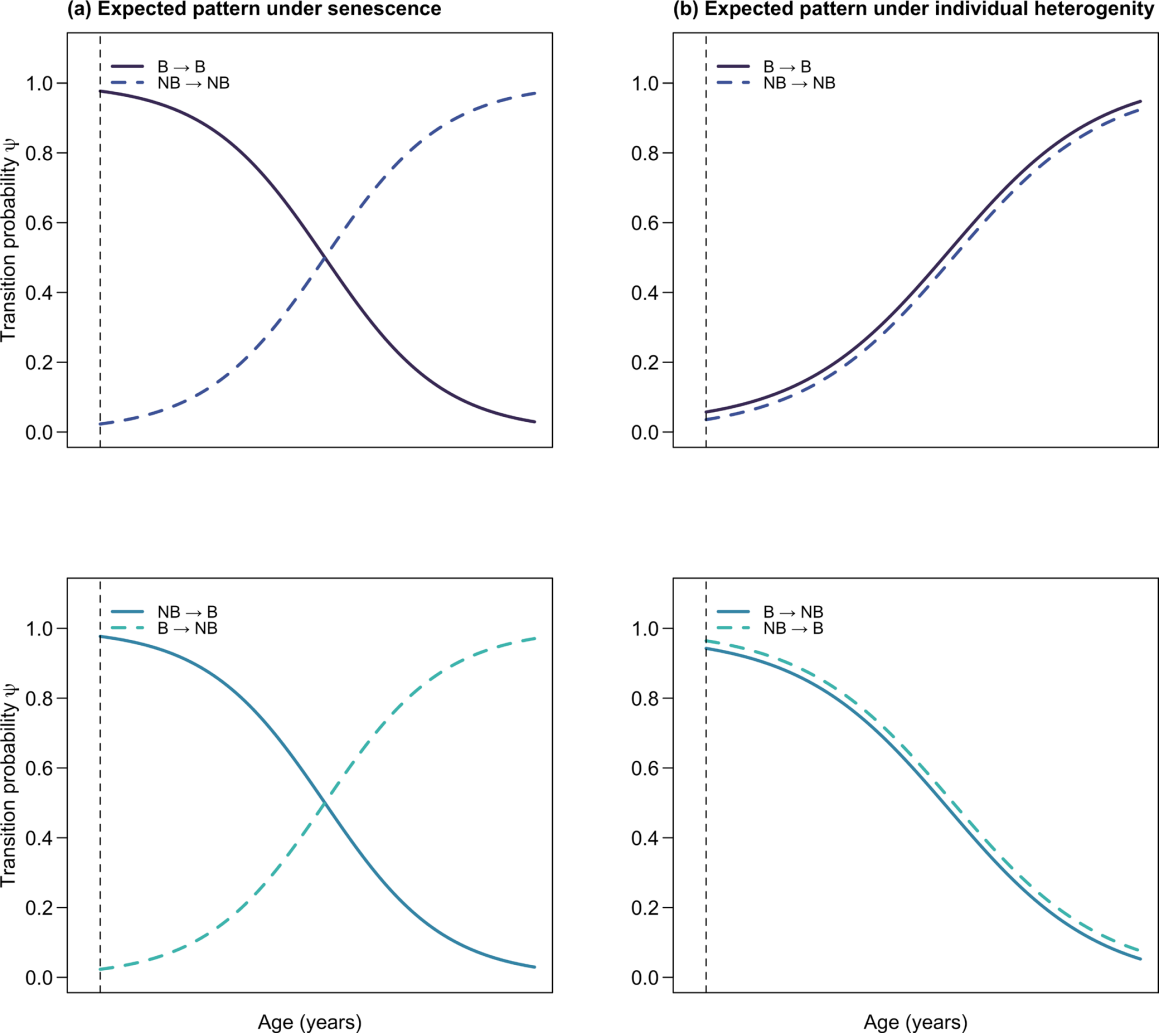
Expected breeding transition patterns under the effect of senescence and individual heterogeneity. If breeding transitions are predominantly influenced by senescence (a), the probability of remaining a breeder (B → B) and transitioning from a non‐breeder to a breeder (NB → B) is expected to decrease with age. Consequently, the probability of remaining a non‐breeder (NB → NB) and transitioning from a breeder to a non‐breeder (B → NB) would then increase with age. Under individual heterogeneity (b), breeders are expected to continue breeding into old age. Similarly, non‐breeders are removed from the population of reproductively active adults. This occurs because of the selective disappearance of less fit individuals, resulting in a more homogeneous pool of experienced breeders at older ages. Vertical dashed lines represent age at maturity.

According to evolutionary theories, the declining force of natural selection with age results in physiological deterioration, which manifests as a change in reproduction and/or survival (Partridge and Barton 1993). Medawar (1952) posited that this weakening in purifying selection allows harmful late‐life mutations to accumulate in the germline. On the other hand, life history optimality models predict that evolutionary trade‐offs and constraints shape aging trajectories (Wachter et al. 2014). For instance, Williams (1957) proposed that pleiotropic alleles with antagonistic effects are maintained in a population when they offer fitness benefits early in life even if they become detrimental later on. Building on work by Hamilton (1966), Kirkwood (1977) frames senescence as an optimal resource allocation problem, whereby reproduction is prioritized over long‐term somatic maintenance. Together, these foundational theories provide a conceptual blueprint to understand the expected relationship between breeding probability and age (Figure 1a), but see Figure 1b for a confounding effect of individual heterogeneity (Gimenez et al. 2018; Wilson and Nussey 2010).

While senescence was once considered an inevitable fate for all organisms (Hamilton 1966), a growing body of research on wild and captive populations is revealing a diverse array of aging patterns across the tree of life (Jones et al. 2013). Declines in survival [actuarial senescence] and reproduction [reproductive senescence] are not uniform across species, populations, individuals, or even traits within individuals (Gaillard and Lemaître 2017; Jones et al. 2013). Organisms with life histories that favor negligible senescence are, therefore, of particular interest to demographers and gerontologists (Jones and Vaupel 2017; Vaupel et al. 2004). Negligible senescence is expected to be common in taxa with protective phenotypes where mortality risk and fecundity are size‐dependent (Vaupel et al. 2004). Under these conditions, natural selection may favor more efficient genetic repair mechanisms to slow the accumulation of DNA and protein damage. One such taxonomic order, testudines (turtles and tortoises), were identified in the 1990s as a prime candidate for exhibiting negligible senescence (Finch 1990). Within reptiles, testudines contain a relatively high number of long‐lived species, earning them recognition as paragons of longevity (Gibbons 1987). A low metabolic rate combined with traits such as an armored shell and continued growth after maturity may have strengthened selection for enhanced somatic repair mechanisms, potentially prolonging fertility into old age. These traits uniquely position testudines to expand current theories of aging (Reinke et al. 2022; da Silva et al. 2022).

Research on the evolution of aging, however, suffers from two systematic biases. First, studies on reproductive aging have traditionally focused on measures of fecundity and fertility, such as offspring number (Nussey et al. 2008). Second, the research that does address breeding probability is taxonomically skewed toward birds (Cam et al. 2002; Jenouvrier et al. 2005; Pradel et al. 2012), mammals (Beauplet et al. 2006; Gamelon et al. 2020; Payne et al. 2024), and amphibians (Cayuela et al. 2014, 2016a). When age is included, rarely does it encompass the entire lifespan of a species (Barbraud and Weimerskirch 2005; Cam and Monnat 2000; Cayuela et al. 2015), but instead is artificially dichotomized into first‐year breeders and all age classes thereafter. As a result, patterns of age‐specific changes in breeding probability, and how it structures reproductive schedules, remain critically understudied in many long‐lived ectotherms, particularly sea turtles (Hoekstra et al. 2020).

As the only extant marine testudines, sea turtles diverged from their terrestrial relatives around 100 million years ago (Bentley et al. 2023). Their unique reproductive niche bridges the gap between highly iteroparous and semelparous life histories. Sea turtles have the largest reproductive output of all oviparous reptiles, yet take decades to mature and undertake extensive reproductive migrations, which lead to extended periods of reproductive quiescence (Broderick et al. 2003). Despite decades of research, no long‐term longitudinal data exist for known‐age sea turtles in natural populations, limiting the use of typical demographic tools in analyzing life history patterns. Here, we leverage an unparalleled 52‐year longitudinal dataset on captive green turtles *Chelonia mydas* to construct a state‐based life history model (McNamara and Houston 1996). We (1) quantify how breeding probability changes across lifespans and determine the key factors driving reproductive schedules, (2) test whether age‐specific changes in breeding probability and survival probability are correlated, and (3) assess the age specificity of changes in demographic properties such as the residual reproductive value (Fisher 1958; Pianka 1976), which is an important indicator of future reproductive potential (Partridge and Barton 1996).

## 2 Methods

### 2.1 Data Collection

The green turtle breeding population at the Cayman Turtle Center (CTC) comprises a mix of captive‐born and wild‐caught animals. The founding population consisted of eggs and adults collected from genetically different nesting and foraging ground aggregations in the Atlantic (Barbanti et al. 2019). For a full description of the founding turtle population and changes in its size over time refer to Barbanti et al. (2019). The breeding population of green turtles is housed in a 2100m^2^ enclosure bordered by an artificial nesting beach. During the nesting season (May to October), staff patrol the beach and collect data on females during oviposition. In addition to the breeding census, a population census is carried out annually, during which turtles in the breeder tank are captured, measured, and re‐tagged (if necessary) using both metal (titanium) flipper and passive integrated transponder (PIT) tags.

Our final dataset included 156 known‐aged females born between 1966 and 2002 with 9 to 45 recaptures per individual (mean = 20.55 ± 7.79 SD). The average number of post‐maturity recaptures per individual was 8.62 (± 8.01 SD). Death was recorded for 22% of the 156 turtles (*n* = 34). However, 21% of the remaining 78% were not observed after Hurricane Michelle in 2001. There were 68 known‐age females still in the breeding tank in 2000. Eighteen of those 68 turtles were encountered after the hurricane, and 45 new breeding females were added after 2002 that were born between 1993 and 2002. As of 2018, 44 of these 63 known‐aged females were still encountered during breeding surveys and population censuses.

### 2.2 Statistical Analysis

#### 2.2.1 Multi‐Event Modeling Framework

To understand factors affecting breeding probability, we built multi‐event capture–mark‐recapture models (MECMR) (Pradel 2005) using E‐SURGE (Choquet and Nogue 2011). MECMR models are defined by three structural parameters: (1) an initial state vector of probabilities π, (2) between‐state transition probabilities, defined by sub‐matrices for survival ϕ and transitions conditional on survival ψ, and (3) event (detection) probabilities p. This highly flexible framework can fit a wide range of complex model structures to account for time, age, and state dependence.

We constructed capture histories where each female was either detected as a juvenile (J; **1**), detected as a subadult (SA; **2**), detected as an adult non‐breeder (NB; **3**), detected as an adult breeder (B; **4**), recovered dead (D; **5**) or not observed (**0**). Detection probabilities were conditional on the first capture, with the initial state vector, π, containing a single element ensuring each turtle is first encountered as a juvenile at birth (age zero; Equation 1). Transitioning between states is represented via a Markov process, where moving to state *i* at time *t* + 1 depends only on the current state. As state transitions are not directly observed, the model adopts a hidden Markov structure. The full state‐dependent transition matrices are shown in Equations (2) and (3). In short, states at time *t* are specified by rows of the survival matrix (Equation 2), and each element of the survival matrix represents the probability of surviving to time *t + 1* in a given state. Likewise, each element of the transition matrix (Equation 3) represents the probability conditional on survival of moving to state *i* at time *t + 1*. The complete transition matrix is the product of ϕ and ψ. Conditional probabilities link observed events to an underlying true state, which may be unknown at the time of capture (Pradel 2005).

To account for heterogeneity in recapture and survival probabilities, as well as in breeding transitions (Péron et al. 2010; Pledger et al. 2003), we implemented a mixture model with two latent states describing individual quality. Reproductively active turtles (states B & NB) were classified as either being of high (superscript +) or low (superscript −) quality. High‐quality females are those which either have a higher survival probability, a higher breeding probability, and/or a higher detection probability relative to low‐quality females. The probability of being in either latent state was determined by observed survival and breeding patterns. The assumption here is that juveniles have a fixed unobserved quality that is constant throughout life, but is only expressed once becoming an adult (Forsythe et al. 2021), that is, transition from a low to high quality state, or vice versa, has a zero probability.

(1) $$\pi =\left(\begin{array}{ccccccc} 1 & 0 & 0 & 0 & 0 & 0 & 0 \end{array}\right)$$

(2) $$\begin{array}{c} \phi =\left(\begin{array}{cccccccc} 1 & 0 & 0 & 0 & 0 & 0 & 0 & 0\\ 0 & 1 & 0 & 0 & 0 & 0 & 0 & 0\\ 0 & 0 & \phi_{\mathrm{NB}}^{-} & 0 & 0 & 0 & 1-\phi_{\mathrm{NB}}^{-} & 0\\ 0 & 0 & 0 & \phi_{\mathrm{NB}}^{+} & 0 & 0 & 1-\phi_{\mathrm{NB}}^{+} & 0\\ 0 & 0 & 0 & 0 & \phi_{B}^{-} & 0 & 1-\phi_{B}^{-} & 0\\ 0 & 0 & 0 & 0 & 0 & \phi_{B}^{+} & 1-\phi_{B}^{+} & 0\\ 0 & 0 & 0 & 0 & 0 & 0 & 0 & 1\\ 0 & 0 & 0 & 0 & 0 & 0 & 0 & 1 \end{array}\right) \end{array}$$

(3) $$\begin{array}{c} \psi =\left(\begin{array}{cccccccc} 1-\left[\psi_{\mathrm{SA}}+\psi_{\mathrm{JB}}^{-}+\psi_{\mathrm{JB}}^{+}\right] & \psi_{\mathrm{SA}} & 0 & 0 & \psi_{\mathrm{JB}}^{-} & \psi_{\mathrm{JB}}^{+} & 0 & 0\\ 0 & 1-\left[\psi_{\mathrm{SAB}}^{-}+\psi_{\mathrm{SAB}}^{+}\right] & 0 & 0 & \psi_{\mathrm{SAB}}^{-} & \psi_{\mathrm{SAB}}^{+} & 0 & 0\\ 0 & 0 & 1-\psi_{B}^{-} & 0 & \psi_{B}^{-} & 0 & 0 & 0\\ 0 & 0 & 0 & 1-\psi_{B}^{+} & 0 & \psi_{B}^{+} & 0 & 0\\ 0 & 0 & \psi_{\mathrm{NB}}^{-} & 0 & 1-\psi_{\mathrm{NB}}^{-} & 0 & 0 & 0\\ 0 & 0 & 0 & \psi_{\mathrm{NB}}^{+} & 0 & 1-\psi_{\mathrm{NB}}^{+} & 0 & 0\\ 0 & 0 & 0 & 0 & 0 & 0 & 1 & 0\\ 0 & 0 & 0 & 0 & 0 & 0 & 0 & 1 \end{array}\right) \end{array}$$

The multi‐event model is structured as follows: Individuals remain juvenile until first observed in the breeder tank, where they transition to a subadult ($\psi_{\mathrm{SA}}$) or a breeder of low ($\psi_{\mathrm{SAB}}^{-}$) or high quality ($\psi_{\mathrm{SAB}}^{+}$). For both juvenile and subadult turtles, survival was set to one since both groups must survive to reproduce at least once. Transitions $\psi_{\mathrm{JB}}$ and $\psi_{\mathrm{SAB}}$ represent transitions from J and SA states to the breeding population. Once mature, individuals alternate between breeding and non‐breeding states until death or the study's end. The probability that a breeder that survived will be absent the following year ($\psi_{\mathrm{NB}}=\psi_{B}\to \psi_{\mathrm{NB}}$) was defined as the departure probability, whereas the arrival probability characterized the probability that an individual absent and surviving will come back to lay eggs the following year ($\psi_{B}=\psi_{\mathrm{NB}}\to \psi_{B}$). E‐SURGE requires users to add the state freshly dead (FD) because recovery can only happen in the year of death. The detection probability p for juvenile turtles was set to one since we only knew the birth year of each individual and when it was first observed in the breeder tank as a subadult (Equation 4). In all other states, individuals can be missed either during the annual census or in a reproductive year.

(4) $$\begin{array}{c} p=\left(\begin{array}{cccccc} 0 & 1 & 0 & 0 & 0 & 0\\ 1-p_{\mathrm{SA}} & 0 & p_{\mathrm{SA}} & 0 & 0 & 0\\ 1-p_{\mathrm{NB}}^{-} & 0 & 0 & p_{\mathrm{NB}}^{-} & 0 & 0\\ 1-p_{\mathrm{NB}}^{+} & 0 & 0 & p_{\mathrm{NB}}^{+} & 0 & 0\\ 1-p_{B}^{-} & 0 & 0 & 0 & p_{B}^{-} & 0\\ 1-p_{B}^{+} & 0 & 0 & 0 & p_{B}^{+} & 0\\ 1-p_{\mathrm{FD}} & 0 & 0 & 0 & 0 & p_{\mathrm{FD}}\\ 1 & 0 & 0 & 0 & 0 & 0 \end{array}\right) \end{array}$$

Transition and survival probabilities were assumed to be constant or vary by age, state, individual quality, or a combination of age, state, and individual quality. Similarly, the encounter probability p was allowed to be constant, or vary by year, state, and individual quality. In models including age effects, $\psi_{\mathrm{SA}}$ was held constant since movement to the breeder tank was not driven by a biological process. When survival was not stratified by breeding state or individual quality, then $\phi =\phi_{\mathrm{NB}}^{-}=\phi_{\mathrm{NB}}^{+}=\phi_{B}^{-}=\phi_{B}^{+}$. If survival was better structured by individual quality, rather than breeding state, then $\phi^{-}=\phi_{\mathrm{NB}}^{-}=\phi_{B}^{-}$ and $\phi^{+}=\phi_{\mathrm{NB}}^{+}=\phi_{B}^{+}$. This parameterization also extends to breeding transitions and encounters, that is, when $p^{-}=p_{\mathrm{NB}}^{-}=p_{B}^{-}$ and $p^{+}=p_{\mathrm{NB}}^{+}=p_{B}^{+}$ recapture probability is structured by individual quality rather than state. Transitions $\psi_{\mathrm{JB}}^{-}$, $\psi_{\mathrm{JB}}^{+}$, $\psi_{\mathrm{SAB}}^{-}$, $\psi_{\mathrm{SAB}}^{+}$, and $\psi_{\mathrm{SA}}$ define recruitment to the breeding population and were constrained to be equal. We refer to this parameter throughout the text as the recruitment probability $\psi_{R}$.

Unfortunately, the multinomial logit‐link function in E‐SURGE does not provide an easy implementation of parametric mortality functions (Ergon et al. 2018), such as those discussed in Gaillard et al. (2004). Since our focus was on changes in breeding probability, we considered this limitation acceptable. For a more detailed assessment of age‐specific mortality in this study system, see Glen et al. (2025). Age was treated as either a continuous variable, defined via a linear‐logistic $\left(\phi_{x}=\frac{\exp\left(\beta_{0}+\beta_{1}\cdot x\right)}{1+\exp\left(\beta_{0}+\beta_{1}\cdot x\right)}\right)$ or a quadratic‐logistic $\left(\phi_{x}=\frac{\exp\left(\beta_{0}+\beta_{1}\cdot x+\beta_{2}\cdot x^{2}\right)}{1+\exp\left(\beta_{0}+\beta_{1}\cdot x+\beta_{2}\cdot x^{2}\right)}\right)$ equation, or a factor (denoted as *a*). Under the linear‐logistic model, annual survival varies monotonically with age with the sign of the slope parameter ($\beta_{1}$) indicating whether survival increases ($\beta_{1}$ > 0) or decreases ($\beta_{1}$ < 0) with age x.

When age was considered a factor, survival, as well as transition parameters were estimated for each age class, although this can lead to parameter identifiability issues at small sample sizes. Therefore, ages 0–6 and 36–45 were binned because (1) no individuals transitioned to the breeding population before age six and (2) less than 5% of turtles were older than 35. We tested whether constraining year‐effects on the encounter probability to known periods of change at the CTC—initial setup (1974–1984) and Hurricane Michelle in 2001—better explained the data than estimating a parameter for each year. This approach assumed that the encounter probability within each $t_{c}$ bin was constant, $t_{c}=\left\{1974-1984,1985-2001,2002-2003,2004-2018\right\}$.

When standard errors for parameter estimates from a fitted model equaled zero, the model was rerun fixing those parameters, and we assessed changes in the deviance. If the deviance was unchanged after fixing a parameter to a constant, we determined that the parameters in question were non‐identifiable. Models were run ten times with random initial values using a quasi‐newton optimization routine to protect against convergence to a local optimum (Lebreton et al. 2009). We used an information criterion‐based approach to assess the relative merit of several hypotheses about changes in survival ϕ and state transitions ψ (Burnham and Anderson 2004). Table S1 lists the tested hypotheses and results. For more information, see SI Appendix S1. The quasi‐Schwarz Information Criterion (QSIC) was calculated as $\frac{\text{deviance}\;}{\hat{c}}+\ln\left(N\right)\cdot K$, where the deviance is $2\cdot \ln\left(\ell \right)$and $\ln\left(\ell \right)$ is the log‐likelihood evaluated at the maximum likelihood (ML) estimates. Differences in QSIC values (∆QSIC) between the best supported model (lowest QSIC) and a competing model were used to evaluate which hypothesis better explained the observed data. We chose a more conservative decision rule of ∆QSIC > 7 units, which indicates strong evidence for one model over another (Jerde et al. 2019).

#### 2.2.2 Goodness‐Of‐Fit Test for the Multi‐Event Model

Before running models in E‐SURGE, goodness‐of‐fit (GOF) tests were performed using a Jolly‐Move Model (JMV) implemented in U‐CARE v3.3 (Choquet et al. 2009). These tests generalize the Arnason‐Schwarz (AS) model and assume that survival, state transitions, and encounter probabilities are homogeneous among individuals (Pradel 2005). Results from the transience tests, which evaluated the null hypothesis that the reencounter probability (3.SR: $\chi^{2}$ = 3.841, *p* = 0.05) and the time until reencounter (3.SM: $\chi^{2}$ = 35.291, *p* = 0.682) for previously and newly encountered turtles were equal, were not significantly different. The WBWA (Where Before Where After) test (Choquet et al. 2009) was significant ($\chi^{2}$ = 109.967, *p* = 0.008), indicating a “memory” effect, or that turtles previously encountered in different states exhibit variation in their expected state when re‐encountered (Pradel et al. 2003). To account for the “memory” effect, we computed the overdispersion coefficient $\hat{c}$, which is the ratio of the Pearson statistic $\chi^{2}$ to its degrees of freedom df, $\hat{c}=\frac{\chi^{2}}{\mathrm{df}}$. The goodness‐of‐fit tests estimated $\hat{c}$ as 1.2636 (149.099/118), which was then used to correct the deviance for the fitted models (see Table S1).

#### 2.2.3 Derived Parameters for the Multi‐Event Model

Following Chevallier et al. (2020) we approximated (1) the inter‐seasonal reproductive period (ISRP), or the interval between reproductive bouts, for surviving turtles $\left(1+\frac{{\psi_{\mathrm{NB}}}_{i}}{{\psi_{B}}_{i}}\right)$, and (2) the probability of a female in a given age‐class *i* having a subsequent reproductive event, $P=\left(1-{\psi_{\mathrm{NB}}}_{i}\right)\cdot \phi_{i}+\frac{{\psi_{\mathrm{NB}}}_{i}\cdot {\psi_{B}}_{i}\cdot \phi_{i}^{2}}{\left(1-\left(1-{\psi_{B}}_{i}\right)\cdot \phi_{i}\right)}$. The expected number of future reproductive events is assumed to follow a geometric distribution with a mean of $\frac{1}{P}$. We also plotted the relationship ϕ and the different breeding transitions, that is, $\psi_{B}^{-}$, $\psi_{B}^{+}$, $\psi_{\mathrm{NB}}^{-}$, and $\psi_{\mathrm{NB}}^{+}$. Variances for derived parameters, including uncertainty in the relationship between ϕ and ψ, were computed using the Delta method.

#### 2.2.4 Calculation of Life History Traits via Matrix Analyses

We also calculated several life history traits using a Leslie matrix constructed from the observed age‐specific fecundity and annual survival estimates derived above. In what follows, we use standard notation for demographic parameters (Salguero‐Gómez and Gamelon 2021).

Let A=U+F, where the Leslie matrix **A** can be decomposed into two matrices relating to survival **U** and fecundity **F**. The sub‐diagonal elements of **A** contained survival estimates for the 46‐age classes (**U**), whereas fecundity (**F**) was the age‐specific mean count of female‐producing eggs, starting from the minimum age at first reproduction (7 years). The raw fecundity data included eggs of both sexes, so we divided the annual number of eggs by two (Wallace et al. 2008; Warden et al. 2015). Assuming a 50:50 sex ratio of eggs is valid because eggs are artificially incubated at temperatures close to the pivotal sex determining temperature—29°C to 30°C (Cayman Turtle Farm 2002; Tilley 2019). Females do not always reproduce annually, so we divided the annual number of female eggs by the population‐level ISRP of 1.7 years (Figure S1). Our primary focus was on population‐level traits, as stratifying populations by measures of individual quality is often impractical. However, we show in the supporting Information' (Figure S2) how varying the ISRP by individual quality captures individual heterogeneity in reproductive traits. Before calculating life history traits (sensu Healy et al. 2019), we verified that **A** was irreducible and ergodic (Caswell 2001).

We first derived the spread of reproduction (or the degree of iteroparity) in captive sea turtles using the Gini index (G). Let $l_{x}m_{x}$ equal fecundity adjusted for survivorship at age x, where $l_{x}$ is the probability of surviving to age x and $m_{x}$ is the maternity function enumerating the number of female eggs per female at age x. Then,

$$G=\frac{2\sum_{x=1}^{n}\left(xl_{x}m_{x}\right)\;}{n\sum_{x=1}^{n}l_{x}m_{x}}-\frac{n+1}{n}$$

A value of G = 1 indicates extreme semelparity (reproduction is isolated to a single event), while G = 0 reflects extreme iteroparity (equal reproduction across all [adult] ages).

To evaluate whether these results align with the mortality and reproductive models in Glen et al. (2025), we computed the shape and pace of aging and fecundity, following Baudisch (2011) and Baudisch and Stott (2019). It is important to note that this analysis uses the same individuals as those in Glen et al. (2025). We used adult life expectancy (denoted as L) to capture the pace of aging, while the pace of fecundity was defined, following Baudisch and Stott (2019), as the age of the mother (minus age at first reproduction, AFR) at oviposition of an average clutch. This measure is the reproductive equivalent of life expectancy in survival analyses (Baudisch and Stott 2019). The shape of aging and fecundity were computed by comparing the area under the survival and cumulative reproductive curves with the area under a function holding reproduction and survival constant. We then assessed the age specificity of changes in reproductive value following section 4.5 of Caswell (2001):

$$\frac{v\left(x\right)}{v\left(0\right)}=\frac{e^{\mathrm{rx}}\;}{l_{x}}\sum_{y=x}^{\infty }\;e^{-\mathrm{ry}}l_{y}m_{y}$$

where $v\left(x\right)$ is the age‐specific reproductive value relative to that of a female newborn ($v\left(0\right)$). Importantly, as shown by Hamilton (1966), $v\left(x\right)$ itself is not a direct measure of the strength of selection at a given age. Rather, as argued by Partridge and Barton (1996), $v\left(x\right)$ is an evolutionarily relevant indicator of an organism's state since it measures the potential of an organism to produce future offspring. Therefore, $v\left(x\right)$ returns the expected future contribution of eggs/offspring to the population at age x. The contribution of offspring to population growth are discounted by a factor of $e^{r}=\lambda$, where r is the Malthusian rate of increase and λ is the population growth rate. Early reproduction is favored in an increasing population ($e^{r}>1$) because offspring born later will contribute less to the gene pool, and vice versa for a decreasing population.

Finally, to estimate average lifetime reproductive output, we computed net reproductive rate ($R_{0}=\sum_{x=1}^{N}\left(l_{x}m_{x}\right)$), or the average number of female eggs produced by a female during her lifetime. By multiplying $R_{0}$ by two, we obtain the net reproductive rate for male and female eggs (total reproductive output). Analyses were conducted using the R package *popbio* (Stubben and Milligan 2007) and *Rage* (Jones et al. 2022). For further methodological details, see Caswell (2001), Healy et al. (2019), and Jones et al. (2022).

#### 2.2.5 Analysis of Inter‐Arrival Times

While Chevallier et al. (2020) provides population‐level estimates for the ISRP, their approach cannot test biological drivers of this interval. Instead, we applied a semi‐parametric Cox proportional hazard model (Landes et al. 2020) to assess how age and reproductive history influence inter‐reproductive timing. This framework estimates (1) the hazard function without making assumptions about the distribution of the baseline hazard rate, $h_{0}\left(\cdot \right)$, and (2) the effects of covariates on the probability of reproducing. The hazard function for the Cox model is expressed as: $h\left(t;x_{i}\right)=h_{0}\left(t\right)e^{x_{i}^{T}\beta }$, where β is a vector of unknown regression parameters related to covariates x for individual *i* at time *t*. We included covariates for age, breeding experience (the number of breeding seasons a female has had so far), AFR, and reproductive effort (the current and cumulative number of clutches or eggs produced). Models were fit using the coxph function in the R package survival version 3.7–0 (Therneau and Grambsch 2002). Correlated groups of observations within an individual were accounted for using a cluster term inside the *coxph* model (Therneau and Grambsch 2002). The proportional hazard assumption, that is, a proportionality of covariate effects over time (Grambsch and Therneau 1994), was tested using the *cox.zph* function and model selection for which covariates best explained the observed data was implemented using the same information criterion‐based approach described above. See SI Appendix S1 for more information. We also fit a generalized linear model (GLM) in the R package *VGAM* (Yee 2015) to the ISRP data. The GLM was fit using a positive Poisson error distribution to ensure that predicted ISRP values are strictly ≥1. We included covariates for the best supported proportional hazards model described above. This approach also allowed us to implement standard regression diagnostics, such as a check for multicollinearity. All analyses were conducted in R version 4.4.1.

## 3 Results

### 3.1 Age‐Specific Changes in Breeding Probability

**FIGURE 2:**
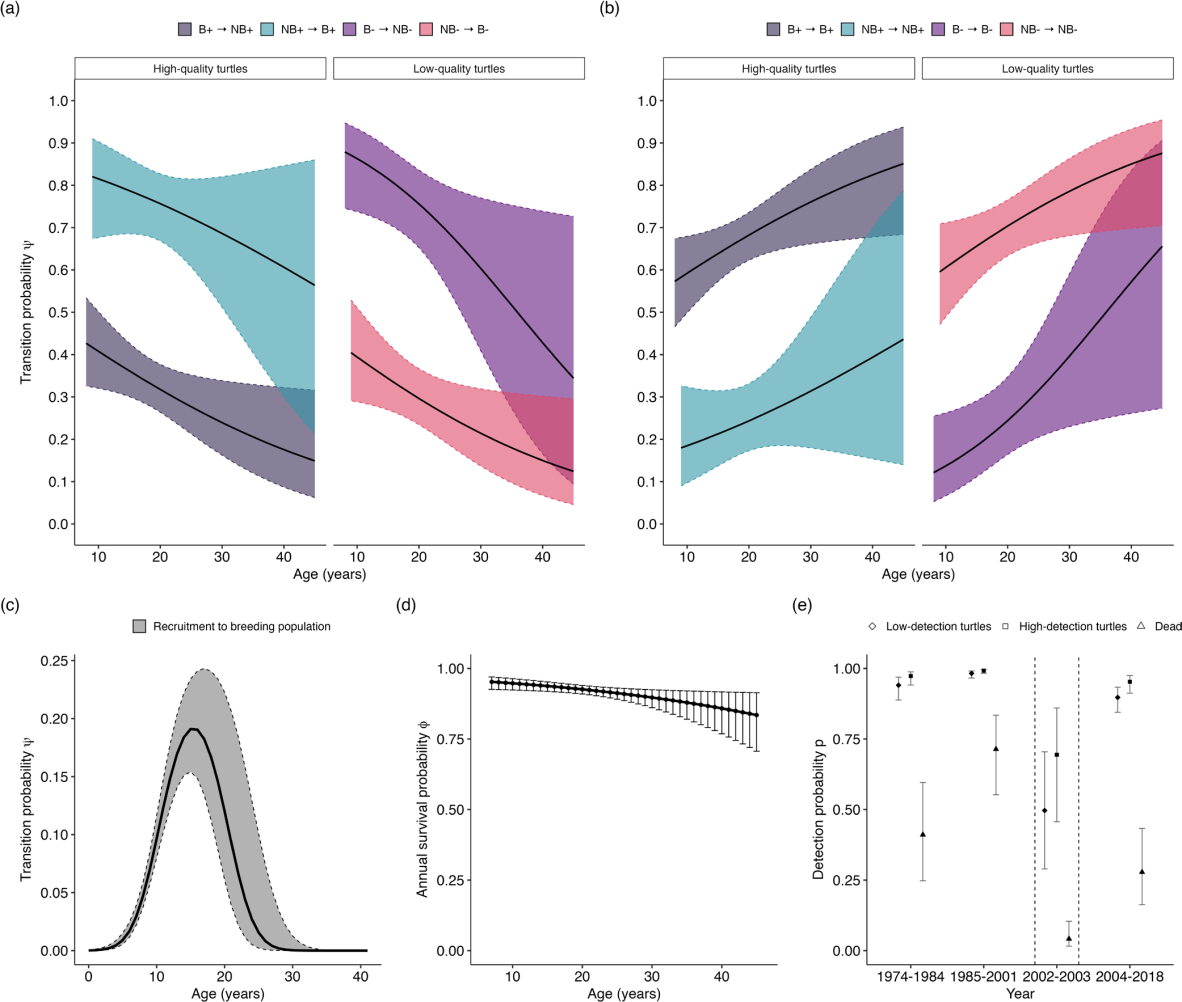
Probability of transitioning between breeding states decreases with age for both latent quality classes. (a, c) Age‐specific state transition probabilities (±95% CI) and (b) age‐specific probability of remaining in the current breeding state (±95% CI). (d) Changes in annual survival probability by age (±95% CI). (e) Detection probabilities by year (±95% CIs). Dashed lines in (e) show changes in the detection probability related to Hurricane Michelle in 2001.

Our 52‐year longitudinal MECMR analysis had 3361 capture histories for 156 captive adult female green turtles. We found that age primarily influenced survival, recruitment, and breeding transitions, outperforming alternative mechanisms in explaining reproductive dynamics (Table S1, Figure 2). As expected, adult survival probability was high (mean: 0.91 [95% CI: 0.87–0.94]), but declined with age ($\beta_{1}$ = −0.49 [95% CI: −0.31 to −0.68]). Survival did not vary by breeding status, breeding status stratified by age, or breeding status stratified by age and individual‐quality (Table S1, Figure 2d). Detection probability was influenced by individual‐quality and year rather than breeding state. Females occasionally evade detection during the annual census, resulting in the detection probability falling below one. Regardless, *p* was relatively high for all states except dead recoveries in non‐hurricane years ($p_{\mathrm{SA}}$ = 0.99–1.00, $p^{-}$ = 0.90–0.98, $p^{+}$ = 0.96–0.99). Dead recoveries ($p_{\mathrm{FD}}$) were lower (0.28–0.71; Figure 2e), likely due to under‐reporting (Barbanti et al. 2019).

**FIGURE 3:**
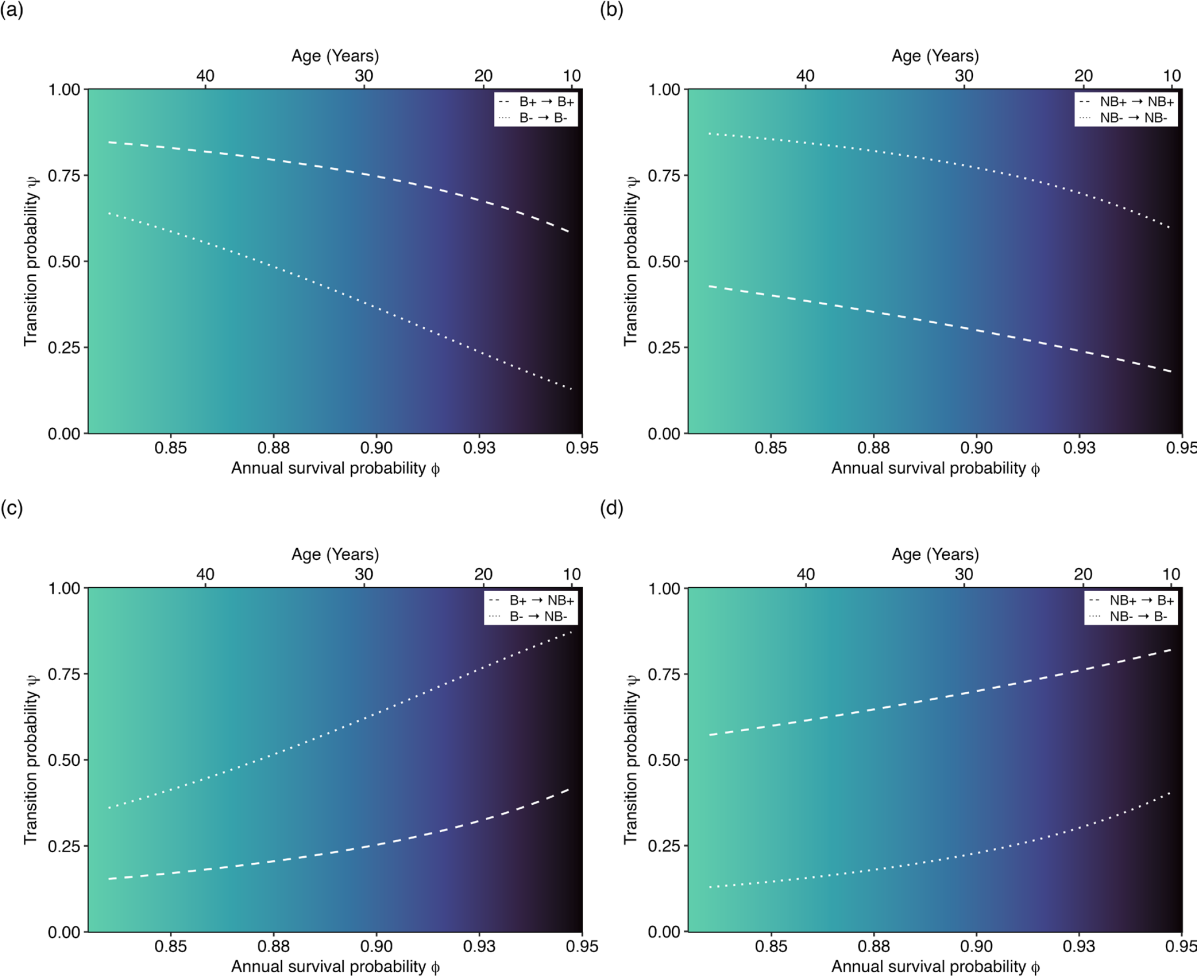
The relationship between annual survival probability and breeding transitions is modulated by age. The figure panel (a–d) displays how transition probabilities, that is, the probability of remaining a breeder (a) or a non‐breeder (b) and transitioning to a breeding (d) or a non‐breeding (c) state, varies with annual survival (bottom x axis). Lines show the fitted mean relationship for low‐quality (dotted lines) and high‐quality (dashed lines) turtles. Background color represents age over a gradient (top *x* axis).

Between‐state transitions were not constant but rather varied with age and individual quality (Figure 2a–c). A quadratic function best modeled the probability of transitioning to the adult (breeding) demographic. The youngest sexually mature individual was 7 years old. At this age, there was a 0.02 (95% CI: 0.01–0.03) probability of becoming an adult (Figure 2c), increasing to 0.19 by age 16 (95% CI: 0.15–0.23). Transitions between breeding and non‐breeding states decreased linearly with age and varied by individual quality. High‐quality turtles consistently had a greater probability of remaining a breeder and a lower probability of becoming a non‐breeder than low‐quality turtles (Figure 2a,b). However, the probability of remaining in their current breeding state increased with age for both groups. For high‐quality turtles, the arrival probability at age 17 was 0.78 (95% CI: 0.69–0.86), decreasing to 0.66 (95% CI: 0.44–0.83) by age 34. The decrease in arrival probability with age was more dramatic for low‐quality turtles. At age 17, low‐quality turtles had an arrival probability of 0.33 (95% CI: 0.26–0.41), decreasing to 0.19 (95% CI: 0.11–0.31) by age 34. By age 40, the probability of remaining in a breeding state ($1-\psi_{\mathrm{NB}}$) was 0.81 (95% CI: 0.68–0.91) for high‐quality turtles and 0.55 (95% CI: 0.26–0.81) for low‐quality turtles, and the probability of remaining in a non‐breeding state ($1-\psi_{B}$) was 0.38 (95% CI: 0.15–0.68) for high‐quality turtles and 0.84 (95% CI: 0.70–0.93) for low‐quality turtles. For correlations between survival and breeding state transitions, see Figure 3.

Importantly, the best supported model via QSIC remained largely unchanged when using the quasi‐Akaike information criterion with small‐sample correction (QAICc). Including parameters by year better represented the data using QAICc than dividing time into blocks where significant demographic changes were expected to occur. Furthermore, QAICc, and by extension QAIC, is an efficient criterion that reduces mean squared prediction error but tends to overfit. In contrast, QSIC is consistent, asymptotically selecting the true model when included in the set of models compared.

Based on estimates for the arrival probability, departure probability, and ϕ, the ISRP was 1.42 years (95% CI: 1.39–1.46) for high‐quality turtles and 3.39 years (95% CI: 3.11–3.67) for low‐quality turtles (Figure S1a), whereas the observed ISRP was 1.7 years. While both groups showed a high probability of a future reproductive event (Figure S1b), for low‐quality individuals, this probability declined more sharply with age. For instance, the probability of reproducing again at age 10 was 0.92 for high‐quality turtles and 0.85 for low‐quality turtles. By age 30, these probabilities dropped to 0.87 and 0.71, respectively. The average probability of a future reproductive event across all adult ages was 0.88 for high‐quality turtles and 0.75 for low‐quality turtles.

### 3.2 Demographic Rates and Aging Patterns

**FIGURE 4:**
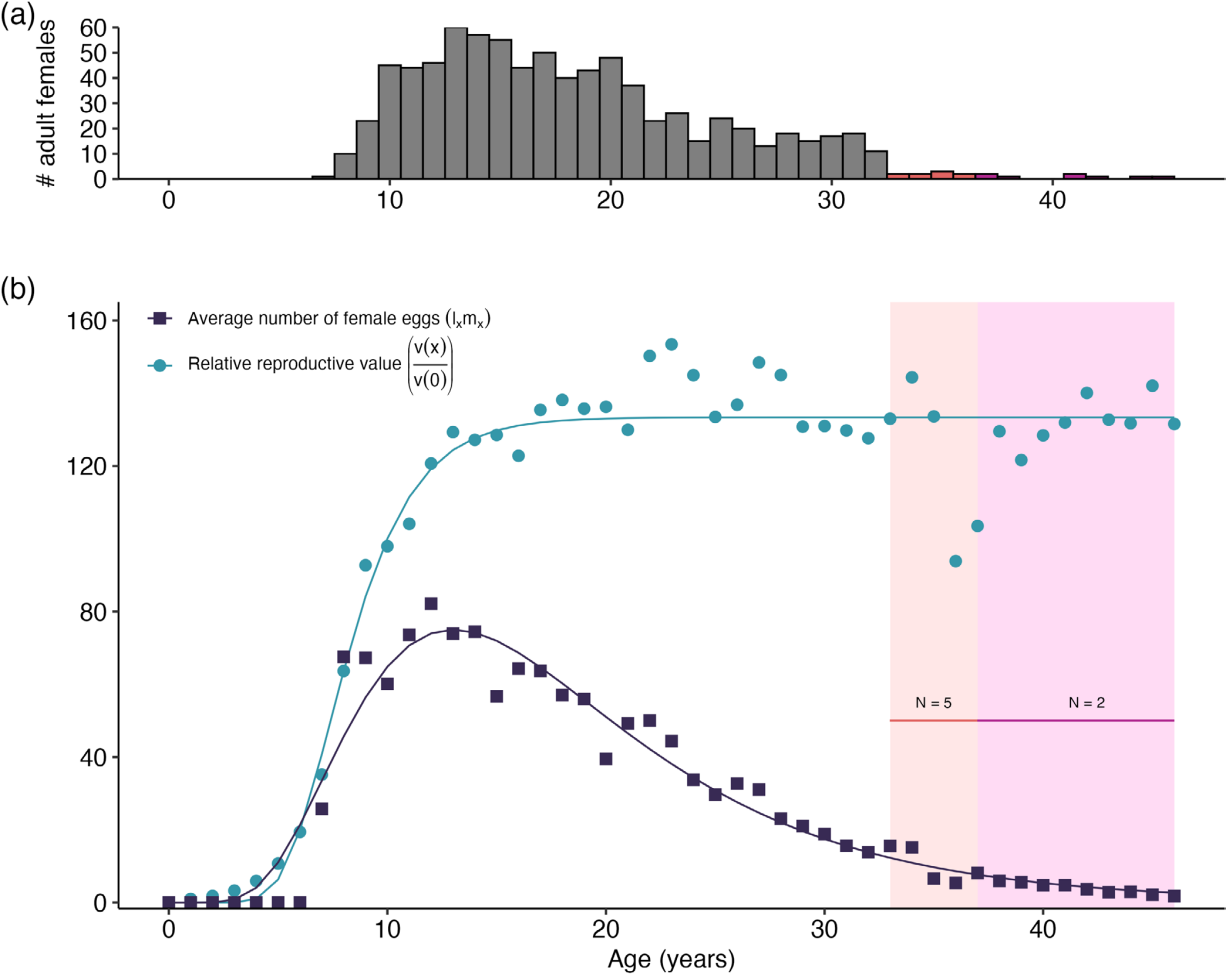
Captive green turtles maintain a high residual reproductive value into old age. (a) Number of adult (mature) females per age class. (b) Age‐specific changes in residual reproductive value and female egg production adjusted for survival ($l_{x}m_{x}$). Curves in (b) represent a *nls* fit for a Gompertz ($l_{x}m_{x}$ data) and lognormal (residual reproductive value data) model.

Adult females had a life expectancy of 20.90 years, with a post‐maturity life expectancy of 13.90 years and a generation time of 12.24 years. Females had a reproductive window of 12.54 years (pace of fecundity) and a moderate degree of iteroparity (Gini index = 0.65). The net reproductive rate ($R_{0}$) was 1427 female eggs per female, totaling an average lifetime fecundity of 2855 eggs. This high fecundity, enhanced by protection in captivity, produced a population growth rate (λ) of 1.81. Analysis of aging revealed low but positive actuarial senescence (shape = 0.16) and negligible reproductive senescence (shape = −0.03). The residual reproductive value remained constant from age 15 onwards (Figure 4).

### 3.3 Effect of Age and Reproductive Effort on Inter‐Arrival Times

**FIGURE 5:**
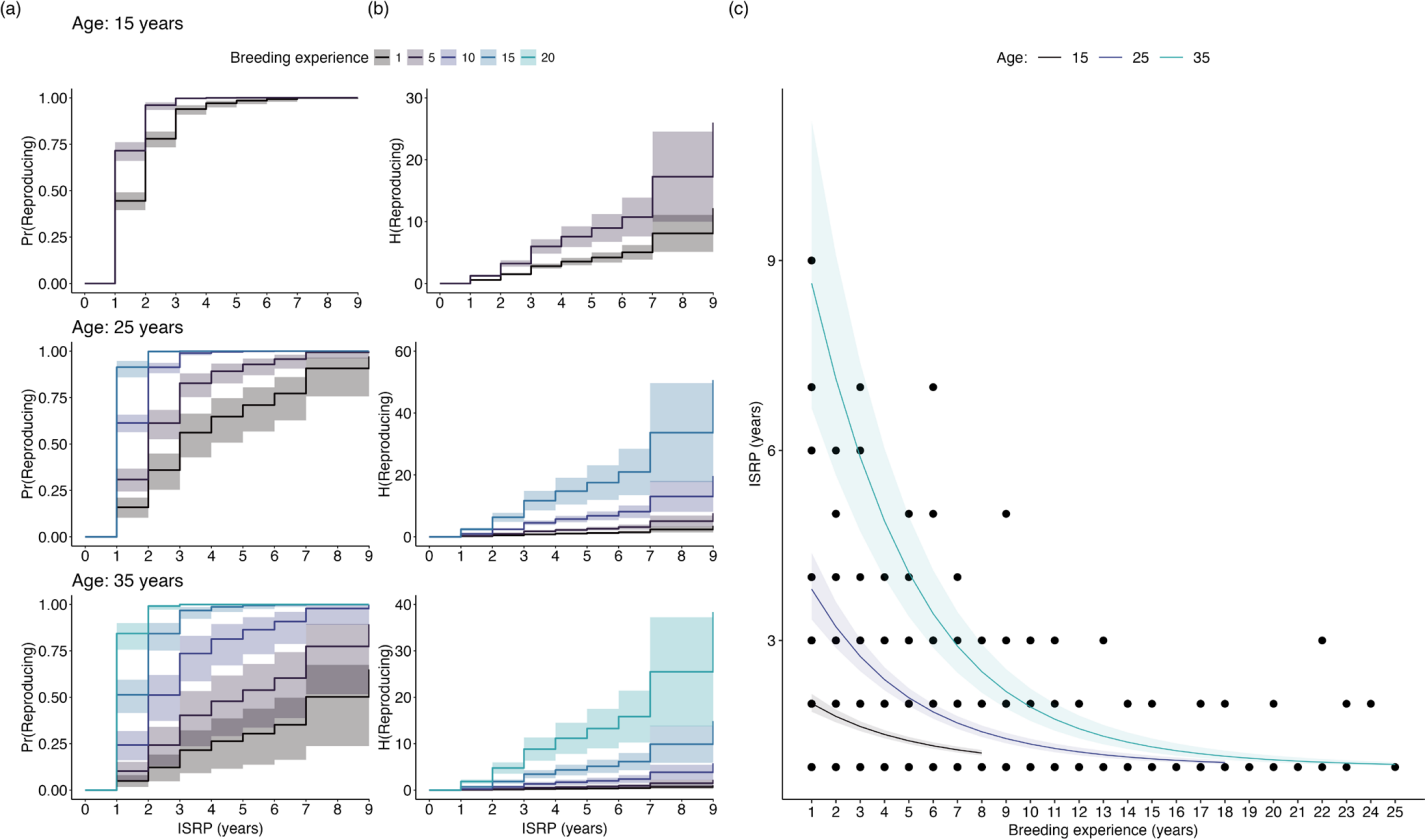
Captive green turtles with more breeding experience wait fewer years between breeding events. (a) Probability of reproducing—$\mathrm{Pr}\left(\text{Reproducing}\right)$—by age and breeding experience over different inter‐seasonal reproductive periods (ISRP). (b) Hazard rate of reproducing per unit time—$H\left(\text{Reproducing}\right)$. (c) Relationship between the ISRP with breeding experience and age. Colored lines are predictions from the best supported model, and shaded areas indicate 95% Wald‐type CIs.

We found strong evidence that age and breeding experience predicted the length of the ISRP (Table S2), rather than additive or multiplicative measures of reproductive effort. Carry‐over effects from reproductive effort did not increase the ISRP. Breeding experience and age had antagonistic effects (Table 1a). These two time‐related measures do not always increase isometrically (Figure 5). As individuals aged, the hazard (risk of reproducing) declined by approximately 11.5% (*p* < 0.001), whereas the hazard rate significantly increased with breeding experience by approximately 21% (*p* < 0.001). These results support the MECMR models, which suggest aging individuals with lower breeding experience are less likely to become reproductively active (Figure 5a,b). Conversely, animals continuing to reproduce tend to have accumulated more breeding experience, and remain reproductively active. The global chi‐square test showed that the proportional hazards assumption was not violated ($\chi_{2}^{2}$ = 0.3667, *p* = 0.83). Results for the GLM were similar to the proportional hazard model (Figure 5c, Table 1b) and we did not find any evidence of multicollinearity (VIF < 2). The ISRP increased by 9.4% per one‐year increase in age (95% CI: 7.3%–10.5%), while it decreased by 15.6% per one‐year increase in breeding experience (95% CI: 13.9%–18.1%).

**TABLE 1:** Effect of age and breeding experience (defined as the number of previous breeding seasons a female had prior to the current reproductive year) on the interval between reproductive years using (a) Cox Proportional Hazard model and (b) a generalized linear model (GLM).

(a)	Estimate (95% CI)	Std. Error	Statistic	*p*
Age	0.88 (0.86–0.91)	0.01	−7.99	< 0.001
Breeding experience	1.21 (1.17–1.25)	0.02	10.28	< 0.001
Observations	662			
Nagelkerke's Pseudo‐*R* ^2^	0.21			
log‐Likelihood	−3563.94			

(b)	Estimate (95% CI)	Std. Error	Statistic	*p*
Intercept	0.55 (0.42–0.71)	0.07	−4.60	< 0.001
Age	1.09 (1.07–1.10)	0.01	12.41	< 0.001
Breeding experience	0.82 (0.80–0.85)	0.01	−13.00	< 0.001
Observations	662			
McFadden's Pseudo‐*R* ^2^	0.13			
log‐Likelihood	−675.32			

*Note:* Values in parentheses are the 95% Wald‐type confidence interval (CI).

## 4 Discussion

Our study revealed three key findings about reproductive traits in captive green turtles. First, the interaction between individual heterogeneity and age primarily drove patterns of breeding probability. The role of reproductive senescence in breeding probability was less clear, whereas individual quality seemed to play a more dominant role in observed reproductive patterns. Interestingly, the probability of remaining in the current breeding state increased with age (Figure 2d). Second, we found no evidence of carry‐over effects from annual or cumulative reproductive effort on the interval between breeding seasons. Instead, breeding experience and age had opposite effects: older, more experienced turtles had shorter intervals between breeding years compared to similar‐aged turtles with fewer prior breeding seasons (Figure 5). Finally, we found that captive green turtles maintain a high residual reproductive value that remains constant into old age (Figure 4). Below, we place these findings in the broader context of life‐history evolution.

### 4.1 The Effect of Age and Individual Heterogeneity on Breeding Probability

In our study, reproductive senescence in the form of a decline in breeding probability is more likely to be expressed in low‐quality turtles. Ignoring individual quality as a factor masked the intensity of this effect. Low‐quality turtles had a reduced probability of breeding and a greater probability of remaining non‐breeders compared to their high‐quality counterparts. High‐quality turtles also had a higher detectability and were more likely to remain reproductively active as they aged, but they did not have a clear survival advantage. Previous work on this population of green turtles reported similar effects of quality on life history trajectories (Glen et al. 2025), that is, even though mortality increased exponentially with age the longest‐lived turtles achieved the largest lifetime reproductive output and reproduced more frequently. Indeed, the demographic analysis via life tables supported these earlier findings, reporting both low but positive actuarial senescence and negligible reproductive senescence at the population level. However, Glen et al. (2025) also reported higher mortality rates among turtles that bred only once relative to those with multiple breeding seasons. Discrepancies in the survival results between the two studies are likely owing to differing statistical approaches. Glen et al. (2025) clustered individuals by reproductive frequency and applied a parametric mortality function to estimate aging rates, whereas, in this study, the focus was on changes in breeding probability. We grouped individuals using a mixture model and then estimated annual survival probability.

Despite theoretical expectations of trade‐offs, positive correlations between survival and reproduction are surprisingly common in nature (Chang et al. 2024; Vedder and Bouwhuis 2018; Van De Walle et al. 2023). The positive relationship between breeding probability and individual quality in our study aligns with patterns across taxa, including ungulates, seabirds, and marine mammals (Le Bohec et al. 2007; Hamel et al. 2009; Lescroël et al. 2009; Reed et al. 2015; Robert et al. 2015; Rotella 2023). For example, in kittiwakes *Rissa tridactyla* (Cam et al. 1998) and subantarctic fur seals *Arctocephalus tropicalis* (Beauplet et al. 2006), non‐breeders were more likely to remain non‐breeders the following year. However, there is a notable taxonomic bias toward mammals and birds with less known about breeding probability in ectotherms.

The few studies that have examined breeding probability in natural sea turtle populations report both reproductive costs (Rivalan et al. 2005) and a role of individual heterogeneity (Kendall et al. 2019). Importantly, neither study was able to include absolute age as a covariate. In leatherback turtles *Dermochelys coriacea* , a higher annual reproductive effort and more frequent reproductive episodes are linked to fewer reproductive seasons over a lifetime (Plot et al. 2012; Rivalan et al. 2005). On the other hand, breeding probability for hawksbill turtles *Eretmochelys imbricata* increased when individuals skipped consecutive reproductive events, but declined thereafter (Kendall et al. 2019). The initial increase in breeding probability is expected because, unlike animals that reproduce annually, reproductive skipping in wild sea turtles is almost obligatory (Prince and Chaloupka 2012). Migration carries a high energetic cost—an expense greatly reduced in captivity. However, access to mates and an abundance of food in captivity can accelerate reproductive schedules that accentuate underlying differences in individual quality. Therefore, while reproductive skipping is crucial for recovering body condition in the wild, in captivity, reproductive skipping does not appear to confer a survival advantage or increase the probability of future reproduction.

As demonstrated across a diverse range of vertebrates, quality effects can shape long‐term demographics via mortality selection (Hawkes et al. 2012). Population size and composition are affected by selection, through a reduction in the representation of low‐quality individuals over time (i.e., selective disappearance effects), and optimization processes, which can improve reproductive performance due to cumulative effects of experience (Congdon et al. 2003). The initial breeding season may act as a strong selective filter acting on a heterogeneous class of inexperienced animals (Nevoux et al. 2007), removing lower‐quality turtles to produce a more homogeneous pool of experienced individuals at older ages. Importantly, phenotypic selection within a cohort can mask reproductive and/or actuarial senescence since any signal of aging may simply be too weak to distinguish from underlying individual heterogeneity (Cam et al. 2002; Hamel et al. 2009)—particularly in captive environments (Ricklefs and Cadena 2007). As a result, disentangling demographic processes from age‐specific changes is notoriously difficult, and failing to detect a trade‐off does not imply its absence (Lemaître et al. 2015; Nussey et al. 2006). This concept was famously illustrated by van Noordwijk and de Jong, who showed that trade‐offs can remain hidden when variation in resource acquisition among individuals exceeds variation in resource allocation (de Jong and van Noordwijk 1992; Metcalf 2016; van Noordwijk and de Jong 1986).

A closer examination of the correlations between survival and transitions among breeding states identified a potential issue with ignoring age as a covariate. For instance, we found no support for modeling survival as state‐dependent (Table S1). Although correlations between survival and breeding transitions suggested lower survival when individuals remained breeders and higher survival associated with transitions to non‐breeders (Figure 3a,c), these patterns did not match predictions of adaptive skipping (Figure 3b,d), which predict that survival rises for consecutive non‐breeders or falls when non‐breeders become breeders. One plausible explanation for this result is that the observed correlations are not evidence of a direct trade‐off, but rather an artifact of a powerful confounding variable: age. As turtles age, both survival probability and the probability of transitioning between breeding states decline. This co‐dependence on age can create a statistical correlation between survival and reproduction. Thus, reproductive costs, if present, appear weak compared with the dominant influence of age and individual heterogeneity. Future studies should be careful when interpreting such correlations as evidence of direct costs without accounting for age effects.

Finally, it is important to note that individual variation in demographic trajectories can manifest as both fixed (e.g., maternal effects) and dynamic heterogeneity (e.g., environmental effects). Dynamic heterogeneity assumes variation in life history trajectories emerges from a first‐order Markov process, such that there is a conditional dependence on transitions between life‐history states. Here, we assumed that the latent grouping of quality was fixed at first reproduction and that transitions between breeding and non‐breeding states were dynamic. This was a reasonable assumption for a captive population where environmental conditions are more stable than in the wild, and our data showed that some individuals consistently outperformed others in reproductive output (Glen et al. 2025). In natural populations, quality may be more dynamic, with individuals transitioning between low‐ and high‐quality states as environmental conditions, such as food quality and predator abundance, fluctuate (Cayuela et al. 2014, 2016b; Muths et al. 2013; Orzack et al. 2011; Öst et al. 2018), and this should be tested and/or accounted for in any modeling framework.

### 4.2 The Degree of Iteroparity in Captive Sea Turtles

Estimates of lifetime reproductive output were similar between this study and Glen et al. (2025)—2602 eggs (Glen et al. 2025) versus 2855 eggs (present study). However, we found that captive green turtles have a moderate degree of iteroparity (*G* = 0.65), mirroring patterns in species such as the eastern mud turtle *Kinosternon subrubrum* (*G* = 0.69) and the Asian elephant *Elephas maximus* (*G* = 0.63) (Healy et al. 2019). In ectotherms, high degrees of iteroparity are associated with slower life histories (Healy et al. 2019), but sea turtles are distinct among reptiles, which may explain this trend. In general, captive green turtles have a high future reproductive potential (Figure 4), but high‐quality females have a 2‐fold increase in their residual reproductive value relative to low‐quality females (Figure S2). As with other sea turtle species, female green turtles have an unusually large intra‐seasonal reproductive effort—producing up to 10 clutches of 120 eggs. Maximizing annual fecundity per reproductive season may, therefore, be a crucial strategy in sea turtles for ensuring maximal lifetime reproductive success.

### 4.3 The Effect of Breeding Experience on the Waiting Time Between Reproductive Years

Beyond age, experience is another critical factor influencing breeding probability (Pradel et al. 2012), though it does not always have a 1:1 relationship with age or age since first reproducing. In line with the multi‐event model results, the probability of reproducing increased with breeding experience. In fact, we found that an effect of individual quality emerged from both the derived ISRP estimates (Figure S1) as well as upon a closer examination of the raw ISRP data (Figure 5). Older turtles with more breeding experience waited fewer years between reproductive events compared to similarly aged turtles with less experience. Comparable results have also been reported in natural populations of testudines (Congdon et al. 2003; Kendall et al. 2019) and mammals (McElligott et al. 2002). As reported in volant (Culina et al. 2019) and non‐volant mammals (Nichols et al. 1994), as well as long‐lived birds (Le Bohec et al. 2007), there was no indication that a higher reproductive effort contributed to longer waiting times. Rather than representing an alternative strategy to enhance residual reproductive value, the degree of non‐breeding and accumulation of breeding experience both seem to be indicators of individual quality driving demographic patterns.

## 5 Conclusions

Negligible actuarial senescence is widespread in captive (da Silva et al. 2022) and wild testudines (Reinke et al. 2022). However, evolutionary theories of aging consider the importance of mortality only as it relates to Darwinian fitness because of knock‐on reproductive effects (Austad and Finch 2022). Similar to Glen et al. (2025), we found evidence of negligible reproductive senescence and low but positive actuarial senescence. However, there was an overarching effect of individual heterogeneity on breeding probability, which aligns with an extensive body of literature on the importance of heterogeneity on broader demographic trends (Forsythe et al. 2021; Gimenez et al. 2018; Wilson and Nussey 2010). Extrapolating our findings to natural sea turtle populations remains speculative, as it is unclear whether they reflect an accelerated reproductive schedule, selection for phenotypes suited to captivity, or both (Farquharson et al. 2018, 2021). These findings are unlikely a result of other factors related to captivity such as inbreeding (*SI* Appendix, S1). Together with Glen et al. (2025), our findings provide comprehensive insight into aging in sea turtles and generate clear, testable hypotheses for future studies on how individual quality, breeding experience, and other life history traits interact to shape demography in wild populations.

## Author Contributions

**C. George Glen:** conceptualization (lead), data curation (lead), formal analysis (lead), investigation (lead), methodology (lead), software (lead), visualization (lead), writing – original draft (lead), writing – review and editing (lead). **Jean‐Dominique Lebreton:** formal analysis (equal), software (supporting), writing – review and editing (supporting). **Walter Mustin:** data curation (supporting), writing – review and editing (supporting). **Karen A. Bjorndal:** funding acquisition (lead), project administration (lead), supervision (lead), writing – review and editing (supporting).

## Conflicts of Interest

The authors declare no conflicts of interest.

## Supporting information


**Data S1:** Supporting Information.

## Data Availability

Third‐party proprietary restrictions prevent public sharing of the raw data under agreements with the Cayman Turtle Center (owners). Access requests may be directed to Species360 or Walter Mustin (wgmustin@turtle.ky) through the Center's research application process, which includes conditions for data use and protection. Raw data are accessible through Research Request applications (form available at \url{https://docs.google.com/forms/d/1znoy62VEkDlhAp_0RfEvF7Zsx03g4W5AlppJHqo3_WQ/viewform?edit_requested=true&pli=1}). The submission of the manuscript under the limitations of the data availability has been approved by the EIC. The submission of the manuscript under the limitations of the data availability has been approved by the EIC.
