# Supplementary material for: Interaction Between Age and Individual Heterogeneity Shapes Breeding Probability in a Long‐Lived Marine Ectotherm
Source: Ecol Evol. 2025 Nov 9;15(11):e72430. doi: 10.1002/ece3.72430 (PMC12597991; doi:10.1002/ece3.72430)
Supplement: Supplementary file 1 — Data S1: Supporting Information. [file ECE3-15-e72430-s001.pdf]

Supplementary Information

Interaction between age and individual heterogeneity shapes breeding probability in a long-lived marine ectotherm

C. George Glen[1,2], Jean-Dominique Lebreton[3], Walter Mustin[4], Karen A. Bjorndal[1,2]

[1]Archie Carr Center for Sea Turtle Research, University of Florida, Gainesville, 32611-8525, FL, USA.
[2]Department of Biology, University of Florida, Gainesville, 32611-8525, FL, USA.
[3]Centre d'Ecologie Fonctionnelle et Evolutive, UMR 5175, CNRS Université de Montpellier, 34293 Montpellier CEDEX 5, France.
[4]Cayman Turtle Center, Grand Cayman, Cayman Islands.

Contributing authors: constantinglen@gmail.com;

1 Supplementary Text

Time-to-event analysis

Time-to-event analysis investigates the probability an event occurs and how that probability changes over time (Landes *et al.*, 2020). This approach accommodates the inclusion of covariates while accounting for censored or truncated data (Klein *et al.*, 2003). In our study, the survival function, $S(t) = Pr(T > t)$, represents the probability an individual will remain reproductively inactive (quiescent) until time t, where t denotes the waiting time to the next reproductive episode and can take values $0 < t < \infty$. At time 0, $S(t)$ equals 1 because females must wait at least one year before reproducing again. Consequently, the probability of reproduction is $1 - S(t)$. The rate of change in the cumulative distribution function $F(t) = Pr(T \leq t)$ is given by the probability distribution function $f(t) = \frac{d}{dt}F(t)$, which can be expressed as the negative rate of change of the survival function, $f(t) = \frac{d}{dt}F(t) = -\frac{d}{dt}S(t)$. The hazard function $h(t) = \lim_{\Delta t \to 0} \frac{Pr(t \leq T < t + \Delta t | T \geq t)}{\Delta t}$ defines the instantaneous risk of reproduction, which can also be expressed as $h(t) = f(t)/S(t)$. Together with the cumulative hazard, $H(t) = \int_0^t h(u)du$, we can directly relate the cumulative hazard and survival function via $S(t) = e^{-H(t)}$.

Model selection using Quasi-Schwarz Information Criterion

Choosing between competing hypothesized data-generating mechanisms is not a trivial task, but requires thoughtful preparation to ensure the scientific arguments under evaluation are accurately translated into statistical statements (Taper & Ponciano, 2016). We used an information criterion-based approach to assess the relative merit of several hypotheses about (1) changes in survival φ and state transitions ψ for the MECMR models and (2) factors affecting the hazard rate in the time-to-event analysis (Burnham & Anderson, 2004; Burnham *et al.*, 2011).

To assess which parameterization provides a better description of the data, we used the Schwarz Information Criterion (SIC; also known as the Bayesian Information Criterion) (Schwarz, 1978). SIC is consistent since the probability of choosing the model in the set

furthest from the generating process goes to zero with increasing sample size (Dennis *et al.*, 2019). The quasi-SIC (QSIC) was computed as $\frac{\text{deviance}}{\hat{c}} + \ln(\text{N}) \cdot \text{K}$, where the deviance is $2\ln(\ell)$ and $\ln(\ell)$ is the log-likelihood evaluated at the maximum likelihood (ML) estimates.

Differences in QSIC values (ΔQSIC) between the best model (lowest QSIC) and a competing model were used to evaluate which hypothesis better explained the observed data. A difference of two units is commonly used as a decision rule since this translates into a 0.05 probability of making the wrong model choice (see Taper & Ponciano (2016)). We chose a more conservative decision rule of $\Delta\text{QSIC} > 7$ units, which suggests strong evidence for one model over another (see Dennis *et al.* (2019) and Jerde *et al.* (2019)). Evidence ratios were also computed to evaluate the level of empirical support for each model in the set (Burnham *et al.*, 2011). The evidence ratio is computed as $\text{ER} = \exp(-(\frac{1}{2} \cdot \Delta\text{QSIC})$.

Comment on genetic variation and adaptation to captivity

Managing the genetic composition of captive populations is crucial to avoid factors affecting individual viability, including founder effects, inbreeding depression, genetic drift, and rapid adaptation to captivity (Farquharson *et al.*, 2021). Captive-born animals often exhibit phenotypic and genotypic changes, sometimes within a single generation, altering fitness (Crates *et al.*, 2023) – a phenomenon known as birth-origin effects (Farquharson *et al.*, 2018). Reduced productivity likely stems from relaxed selection pressures, allowing less fit individuals to survive and reproduce, lowering overall trait values. Non-genetic factors like stress and nutrition can compound these effects. However, species respond to captivity in diverse ways, with some adapting more successfully than others. We do not believe that such genetic issues are a critical problem in this study. Previous research on captive green turtles at the CTC revealed the founder stock – comprising adults and eggs from various nesting beaches and foraging grounds around the Caribbean and Atlantic – initially showed high genetic diversity coupled with a low relatedness (Barbanti *et al.*, 2019). Subsequent cohorts, however, exhibited reduced genetic viability and higher relatedness – an expected consequence of genetic drift in captive breeding programs. This trend stemmed from reliance on individuals born in 1995 to replenish the population after

Hurricane Michelle (Cayman Turtle Farm, 2002). Our results primarily reflect the genetic composition of the initial diverse, unrelated founder stock rather than inbreeding effects, as most animals in our study originated from wild eggs or offspring born from wild-caught adults prior to 1995. Furthermore, the admixture of genetically differentiated populations can reduce offspring fitness and reproductive capacity through outbreeding depression (Weeks *et al.*, 2011), disrupting local adaptations and epistatic interactions. Outbreeding depression is not a relevant factor since females at the CTC seem to be fully capable of reproduction (Barbanti *et al.*, 2019), displaying vital rates comparable to wild populations (Broderick *et al.*, 2003). Additional research suggests that testudines may adapt well to captivity, as offspring survival in radiated tortoises (*Astrochelys radiata*) and western swamp tortoises (*Pseudemydura umbrina*) remained stable or increased across generations Farquharson *et al.* (2021).

Literature Cited

Barbanti, A., Martin, C., Blumenthal, J.M., Boyle, J., Broderick, A.C., Collyer, L., Ebanks-Petrie, G., Godley, B.J., Mustin, W., Ordóñez, V. *et al.* (2019). How many came home? evaluating ex situ conservation of green turtles in the cayman islands. *Molecular ecology*, 28, 1637–1651.

Broderick, A.C., Glen, F., Godley, B.J. & Hays, G.C. (2003). Variation in reproductive output of marine turtles. *Journal of Experimental Marine Biology and Ecology*, 288, 95–109.

Burnham, K.P. & Anderson, D.R. (2004). Multimodel inference: understanding aic and bic in model selection. *Sociological methods & research*, 33, 261–304.

Burnham, K.P., Anderson, D.R. & Huyvaert, K.P. (2011). Aic model selection and multimodel inference in behavioral ecology: some background, observations, and comparisons. *Behavioral ecology and sociobiology*, 65, 23–35.

Cayman Turtle Farm (2002). Application to register a captive breeding operation involving chelonia mydas on grand cayman, cayman islands. Submitted by the CITES Management Authority of the United Kingdom of Great Britain and Northern Ireland

pursuant to Conference Resolution, 11, 14.

Crates, R., Stojanovic, D. & Heinsohn, R. (2023). The phenotypic costs of captivity. *Biological Reviews*, 98, 434–449.

Dennis, B., Ponciano, J.M., Taper, M.L. & Lele, S.R. (2019). Errors in statistical inference under model misspecification: evidence, hypothesis testing, and aic. *Frontiers in Ecology and Evolution*, 7, 372.

Farquharson, K.A., Hogg, C.J. & Grueber, C.E. (2018). A meta-analysis of birth-origin effects on reproduction in diverse captive environments. *Nature Communications*, 9, 1055.

Farquharson, K.A., Hogg, C.J. & Grueber, C.E. (2021). Offspring survival changes over generations of captive breeding. *Nature Communications*, 12, 3045.

Jerde, C.L., Kraskura, K., Eliason, E.J., Csik, S.R., Stier, A.C. & Taper, M.L. (2019). Strong evidence for an intraspecific metabolic scaling coefficient near 0.89 in fish. *Frontiers in Physiology*, 10, 1166.

Klein, J.P., Moeschberger, M.L. *et al.* (2003). *Survival analysis: techniques for censored and truncated data.* vol. 1230. Springer.

Landes, J., Engelhardt, S.C. & Pelletier, F. (2020). An introduction to event history analyses for ecologists. *Ecosphere*, 11, e03238.

Schwarz, G. (1978). Estimating the dimension of a model. *The annals of statistics*, pp. 461–464.

Taper, M.L. & Ponciano, J.M. (2016). Evidential statistics as a statistical modern synthesis to support 21st century science. *Population Ecology*, 58, 9–29.

Weeks, A.R., Sgro, C.M., Young, A.G., Frankham, R., Mitchell, N.J., Miller, K.A., Byrne, M., Coates, D.J., Eldridge, M.D., Sunnucks, P. *et al.* (2011). Assessing the benefits and risks of translocations in changing environments: a genetic perspective. *Evolutionary Applications*, 4, 709–725.

2 Implementation of Capture-Mark-Recapture models in E-SURGE

To implement the models in E-SURGE, we had to define a pattern (step GEPAT) and a structure (Step GEMACO) of parameters. The input for the final model (model XXXX from Table S1) in the paper is presented as follows:

```
E-SURGE V 2.2.3,23-Jul-2025

Number of occasions :  53
Number of states :   8
Number of events :   6
Number of groups :   1
Number of age classes :  46
~~~~~~~~~~~~~~~~~~~~~~~~~~~~~~~~~~~~~~~~~~~~~~~~~~~~~~~~~~~~~~~~~~~~~~
Model formula (or file) :
 For Initial State:
 IS - Step 1 - (0): i
 For Transition:
 S - Step 1 - (2): linearage
 For Transition:
 T - Step 2 - (12): from(1).to(2) + from(1 2).to(5 6).quadage +
        [from(3, 5).to(3, 5) + from(4, 6).to(4, 6)].linearage
 For Event:
 E - Step 1 - (9): firste + nexte.from(2 ,3 5, 4 6, 7) +
       nexte.constrainedTatE
~~~~~~~~~~~~~~~~~~~~~~~~~~~~~~~~~~~~~~~~~~~~~~~~~~~~~~~~~~~~~~~~~~~~~~
<B>Full Model Details</B>
--------------------
# of step for initial state :1
Phrase for step 1 : i
Number of shortcuts : 1
Pattern matrix :
    *   -   -   -   -   -   -

# of step for transition : 2
Phrase for step 1 : linearage
Number of shortcuts : 1
linearage ->   [  [i + a*x(1)] ]
Pattern matrix :
  * - - - - - - -
  - * - - - - - -
  - - y - - - * -
  - - - y - - * -
  - - - - y - * -
  - - - - - y * -
  - - - - - - - *
  - - - - - - - *
Phrase for step 2 : from(1).to(2) + from(1 2).to(5 6).quadage +
          [from(3, 5).to(3, 5) + from(4, 6).to(4, 6)].linearage
Number of shortcuts : 2
linearage ->   [  [i + a*x(1)] ]
quadage ->   [  [i + a*x(1) + a*x(2)] ]
Pattern matrix :
  * a - - b c - -
  - * - - d e - -
  - - * - f - - -
  - - - * - g - -
  - - h - * - - -
  - - - i - * - -
  - - - - - - * -
  - - - - - - - *

# of step for encounter : 1
```

```
Phrase for step 1 : firste + nexte.from(2 ,3 5, 4 6, 7) +
           nexte.constrainedTatE
Number of shortcuts : 1
constrainedTatE ->   [time(1:8, 9:19, 20:36, 37:38, 39:53)]
Pattern matrix :
  -   *   -   -   -   -
  *   -   b   -   -   -
  *   -   -   b   -   -
  *   -   -   b   -   -
  *   -   -   -   b   -
  *   -   -   -   b   -
  *   -   -   -   -   b
  *   -   -   -   -   -
~~~~~~~~~~~~~~~~~~~~~~~~~~~~~~~~~~~~~~~~~~~~~~~~~~~~~~~~~~~~~~~~~~~~~~
```

3 Supplementary tables

Table S1: Model comparisons results for state-dependent survival and transitions

#	**Hypothesis**<br>Model formula	**Statistical results**<br># Par	Dev	QSIC	ΔQSIC	Evidence ratio[a]
1	$\pi(\cdot)$, $\varphi(\mathrm{A})$, $\psi^{\mathrm{SA}} + [\psi^{\mathrm{R}} \cdot \mathrm{A} + \mathrm{A}^2] + [\psi^{\mathrm{B,NB}} \cdot \mathrm{H} \cdot \mathrm{A}]$, $\mathrm{p}(\mathrm{t_c} + \mathrm{H})$	21	4591.36	3804.07	**0.00**	1
2	$\pi(\cdot)$, $\varphi(\mathrm{A})$, $\psi^{\mathrm{SA}} + [\psi^{\mathrm{R}} \cdot \mathrm{A} + \mathrm{A}^2] + [\psi^{\mathrm{B,NB}} \cdot \mathrm{H} \cdot \mathrm{A}]$, $\mathrm{p}(\mathrm{t_c} + \mathrm{state})$	21	4594.12	3806.26	2.18	0.34
3	$\pi(\cdot)$, $\varphi(\mathrm{A} \cdot \mathrm{H})$, $\psi^{\mathrm{SA}} + [\psi^{\mathrm{R}} \cdot \mathrm{A} + \mathrm{A}^2] + [\psi^{\mathrm{B,NB}} \cdot \mathrm{H} \cdot \mathrm{A}]$, $\mathrm{p}(\mathrm{t_c} + \mathrm{H})$	23	4582.05	3812.95	8.88	0.01
4	$\pi(\cdot)$, $\varphi(\mathrm{A} \cdot \mathrm{H})$, $\psi^{\mathrm{SA}} + [\psi^{\mathrm{R}} \cdot \mathrm{A} + \mathrm{A}^2] + [\psi^{\mathrm{B,NB}} \cdot \mathrm{H} \cdot \mathrm{A}]$, $\mathrm{p}(\mathrm{t_c} + \mathrm{state})$	23	4585.21	3815.44	11.37	‡
5	$\pi(\cdot)$, $\varphi(\mathrm{A})$, $\psi^{\mathrm{SA}} + [\psi^{\mathrm{R}} \cdot \mathrm{A} + \mathrm{A}^2] + [\psi^{\mathrm{B,NB}} \cdot \mathrm{H} \cdot \mathrm{A}]$, $\mathrm{p}(\mathrm{t_c} + \mathrm{state} + \mathrm{H})$	23	4589.20	3818.61	14.53	‡
6	$\pi(\cdot)$, $\varphi(\mathrm{state} \cdot \mathrm{A} \cdot \mathrm{H})$, $\psi^{\mathrm{SA}} + [\psi^{\mathrm{R}} \cdot \mathrm{A} + \mathrm{A}^2] + [\psi^{\mathrm{B,NB}} \cdot \mathrm{H} \cdot \mathrm{A}]$, $\mathrm{p}(\mathrm{t_c} + \mathrm{state})$	27	4578.19	3842.37	38.30	‡
7	$\pi(\cdot)$, $\varphi(\mathrm{A})$, $\psi^{\mathrm{SA}} + [\psi^{\mathrm{R}} \cdot \mathrm{A} + \mathrm{A}^2] + [\psi^{\mathrm{B,NB}} \cdot \mathrm{A}]$, $\mathrm{p}(\mathrm{t_c} + \mathrm{H})$	17	4686.60	3846.96	42.89	‡
8	$\pi(\cdot)$, $\varphi(\mathrm{A} \cdot \mathrm{H})$, $\psi^{\mathrm{SA}} + [\psi^{\mathrm{R}} \cdot \mathrm{A} + \mathrm{A}^2] + [\psi^{\mathrm{B,NB}} \cdot \mathrm{A}]$, $\mathrm{p}(\mathrm{t_c} + \mathrm{H})$	19	4672.29	3851.88	47.81	‡
9	$\pi(\cdot)$, $\varphi(\mathrm{state} \cdot \mathrm{A} \cdot \mathrm{H})$, $\psi^{\mathrm{SA}} + [\psi^{\mathrm{R}} \cdot \mathrm{A} + \mathrm{A}^2] + [\psi^{\mathrm{B,NB}} \cdot \mathrm{H} \cdot \mathrm{A}]$, $\mathrm{p}(\mathrm{t_c} + \mathrm{state} + \mathrm{H})$	29	4574.97	3856.06	51.99	‡
10	$\pi(\cdot)$, $\varphi(\mathrm{A})$, $\psi^{\mathrm{SA}} + [\psi^{\mathrm{R}} \cdot \mathrm{A} + \mathrm{A}^2] + [\psi^{\mathrm{B,NB}} \cdot \mathrm{A}]$, $\mathrm{p}(\mathrm{t_c} + \mathrm{state} + \mathrm{H})$	19	4678.10	3856.48	52.41	‡
11	$\pi(\cdot)$, $\varphi(\mathrm{A} \cdot \mathrm{H})$, $\psi^{\mathrm{SA}} + [\psi^{\mathrm{R}} \cdot \mathrm{A} + \mathrm{A}^2] + [\psi^{\mathrm{B,NB}} \cdot \mathrm{A}]$, $\mathrm{p}(\mathrm{t_c} + \mathrm{state})$	19	4701.95	3875.36	71.28	‡
12	$\pi(\cdot)$, $\varphi(\mathrm{state} \cdot \mathrm{A} \cdot \mathrm{H})$, $\psi^{\mathrm{SA}} + [\psi^{\mathrm{R}} \cdot \mathrm{A} + \mathrm{A}^2] + [\psi^{\mathrm{B,NB}} \cdot \mathrm{A}]$, $\mathrm{p}(\mathrm{t_c} + \mathrm{state} + \mathrm{H})$	25	4649.38	3882.47	78.39	‡
13	$\pi(\cdot)$, $\varphi(\mathrm{state} \cdot \mathrm{A} \cdot \mathrm{H})$, $\psi^{\mathrm{SA}} + [\psi^{\mathrm{R}} \cdot \mathrm{A} + \mathrm{A}^2] + [\psi^{\mathrm{B,NB}} \cdot \mathrm{A}]$, $\mathrm{p}(\mathrm{t_c} + \mathrm{state})$	23	4686.14	3895.32	91.25	‡
14	$\pi(\cdot)$, $\varphi(\mathrm{A})$, $\psi^{\mathrm{SA}} + [\psi^{\mathrm{R}} \cdot \mathrm{A} + \mathrm{A}^2] + [\psi^{\mathrm{B,NB}} \cdot \mathrm{A}]$, $\mathrm{p}(\mathrm{t_c} + \mathrm{state})$	17	4716.39	3870.54	66.47	‡
15	$\pi(\cdot)$, $\varphi(\mathrm{A})$, $\psi^{\mathrm{SA}} + [\psi^{\mathrm{R}} \cdot \mathrm{A} + \mathrm{A}^2] + [\psi^{\mathrm{B,NB}} \cdot \mathrm{A}]$, $\mathrm{p}(\mathrm{t_c} \cdot \mathrm{state})$	26	4661.93	3900.52	96.45	‡
16	$\pi(\cdot)$, $\varphi(\mathrm{A})$, $\psi^{\mathrm{SA}} + [\psi^{\mathrm{R}} \cdot \mathrm{A} + \mathrm{A}^2] + [\psi^{\mathrm{B,NB}} \cdot \mathrm{A}]$, $\mathrm{p}(\mathrm{state})$	14	4799.68	3912.09	108.02	‡
17	$\pi(\cdot)$, $\varphi(\mathrm{A} + \mathrm{A}^2)$, $\psi^{\mathrm{SA}} + [\psi^{\mathrm{R}} \cdot \mathrm{A} + \mathrm{A}^2] + [\psi^{\mathrm{B,NB}} \cdot \mathrm{A}]$, $\mathrm{p}(\mathrm{state})$	15	4794.34	3915.99	111.92	‡
18	$\pi(\cdot)$, $\varphi(\mathrm{state} \cdot \mathrm{A})$, $\psi^{\mathrm{SA}} + [\psi^{\mathrm{R}} \cdot \mathrm{A} + \mathrm{A}^2] + [\psi^{\mathrm{B,NB}} \cdot \mathrm{A}]$, $\mathrm{p}(\mathrm{state})$	15	4797.94	3918.84	114.77	‡
19	$\pi(\cdot)$, $\varphi(\mathrm{A})$, $\psi^{\mathrm{SA}} + [\psi^{\mathrm{R}} \cdot \mathrm{A} + \mathrm{A}^2] + [\psi^{\mathrm{B,NB}} \cdot \mathrm{A} + \mathrm{A}^2]$, $\mathrm{p}(\mathrm{state})$	16	4796.03	3925.45	121.37	‡
20	$\pi(\cdot)$, $\varphi(\mathrm{A} + \mathrm{A}^2)$, $\psi^{\mathrm{SA}} + [\psi^{\mathrm{R}} \cdot \mathrm{A} + \mathrm{A}^2] + [\psi^{\mathrm{B,NB}} \cdot \mathrm{A} + \mathrm{A}^2]$, $\mathrm{p}(\mathrm{state})$	17	4790.70	3929.35	125.28	‡
21	$\pi(\cdot)$, $\varphi(\mathrm{A})$, $\psi^{\mathrm{SA}} + [\psi^{\mathrm{R}} \cdot \mathrm{a}] + [\psi^{\mathrm{B,NB}} \cdot \mathrm{A}]$, $\mathrm{p}(\mathrm{state})$	27	4731.58	3963.76	159.69	‡
22	$\pi(\cdot)$, $\varphi(\mathrm{A})$, $\psi^{\mathrm{SA}} + [\psi^{\mathrm{R}} \cdot \mathrm{A} + \mathrm{A}^2] + [\psi^{\mathrm{B,NB}} \cdot \mathrm{A}]$, $\mathrm{p}(\mathrm{t})$	31	4703.57	3974.08	170.01	‡
23	$\pi(\cdot)$, $\varphi(\mathrm{A} + \mathrm{A}^2)$, $\psi^{\mathrm{SA}} + [\psi^{\mathrm{R}} \cdot \mathrm{a}] + [\psi^{\mathrm{B,NB}} \cdot \mathrm{A}]$, $\mathrm{p}(\mathrm{state})$	29	4726.25	3975.79	171.71	‡
24	$\pi(\cdot)$, $\varphi(\cdot)$, $\psi^{\mathrm{SA}} + [\psi^{\mathrm{R}} \cdot \mathrm{A}] + [\psi^{\mathrm{B,NB}} \cdot \mathrm{A}]$, $\mathrm{p}(\mathrm{state})$	12	4905.49	3979.59	175.52	‡
25	$\pi(\cdot)$, $\varphi(\mathrm{A})$, $\psi^{\mathrm{SA}} + [\psi^{\mathrm{R}} \cdot \mathrm{A}] + [\psi^{\mathrm{B,NB}} \cdot \mathrm{A}]$, $\mathrm{p}(\mathrm{state})$	13	4899.53	3983.00	178.93	‡
26	$\pi(\cdot)$, $\varphi(\mathrm{state})$, $\psi^{\mathrm{SA}} + [\psi^{\mathrm{R}} \cdot \mathrm{A}] + [\psi^{\mathrm{B,NB}} \cdot \mathrm{A}]$, $\mathrm{p}(\mathrm{state})$	13	4903.22	3985.92	181.84	‡
27	$\pi(\cdot)$, $\varphi(\mathrm{A} + \mathrm{A}^2)$, $\psi^{\mathrm{SA}} + [\psi^{\mathrm{R}} \cdot \mathrm{A}] + [\psi^{\mathrm{B,NB}} \cdot \mathrm{A}]$, $\mathrm{p}(\mathrm{state})$	14	4894.20	3986.90	182.82	‡
28	$\pi(\cdot)$, $\varphi(\mathrm{A} \cdot \mathrm{state})$, $\psi^{\mathrm{SA}} + [\psi^{\mathrm{R}} \cdot \mathrm{A}] + [\psi^{\mathrm{B,NB}} \cdot \mathrm{A}]$, $\mathrm{p}(\mathrm{state})$	15	4897.65	3997.75	193.67	‡
29	$\pi(\cdot)$, $\varphi(\mathrm{A} + \mathrm{A}^2 \cdot \mathrm{state})$, $\psi^{\mathrm{SA}} + [\psi^{\mathrm{R}} \cdot \mathrm{A}] + [\psi^{\mathrm{B,NB}} \cdot \mathrm{A}]$, $\mathrm{p}(\mathrm{state})$	17	4889.50	4007.54	203.46	‡
30	$\pi(\cdot)$, $\varphi(\mathrm{A})$, $\psi^{\mathrm{SA}} + [\psi^{\mathrm{R}} \cdot \mathrm{A} + \mathrm{A}^2] + [\psi^{\mathrm{B,NB}} \cdot \mathrm{A}]$, $\mathrm{p}(\mathrm{t} + \mathrm{state})$	58	4500.60	4032.69	228.61	‡
31	$\pi(\cdot)$, $\varphi(\mathrm{a})$, $\psi^{\mathrm{SA}} + [\psi^{\mathrm{R}} \cdot \mathrm{A} + \mathrm{A}^2] + [\psi^{\mathrm{B,NB}} \cdot \mathrm{A}]$, $\mathrm{p}(\mathrm{state})$	37	4745.74	4056.17	252.09	‡
32	$\pi(\cdot)$, $\varphi(\mathrm{A})$, $\psi^{\mathrm{SA}} + [\psi^{\mathrm{R}} \cdot \mathrm{A} + \mathrm{A}^2] + [\psi^{\mathrm{B,NB}} \cdot \mathrm{A}]$, $\mathrm{p}(\mathrm{t_c})$	14	4984.15	4058.08	254.01	‡
33	$\pi(\cdot)$, $\varphi(\mathrm{a})$, $\psi^{\mathrm{SA}} + [\psi^{\mathrm{R}} \cdot \mathrm{A} + \mathrm{A}^2] + [\psi^{\mathrm{B,NB}} \cdot \mathrm{A} + \mathrm{A}^2]$, $\mathrm{p}(\mathrm{state})$	39	4742.09	4069.52	265.45	‡
34	$\pi(\cdot)$, $\varphi(\mathrm{a})$, $\psi^{\mathrm{SA}} + [\psi^{\mathrm{R}} \cdot \mathrm{a}] + [\psi^{\mathrm{B,NB}} \cdot \mathrm{A}]$, $\mathrm{p}(\mathrm{state})$	50	4677.69	4107.87	303.80	‡
35	$\pi(\cdot)$, $\varphi(\cdot)$, $\psi^{\mathrm{SA}} + \psi^{\mathrm{R}} + \psi^{\mathrm{B,NB}}$, $\mathrm{p}(\mathrm{state})$	9	5100.28	4109.39	305.32	‡
36	$\pi(\cdot)$, $\varphi(\mathrm{A})$, $\psi^{\mathrm{SA}} + \psi^{\mathrm{R}} + \psi^{\mathrm{B,NB}}$, $\mathrm{p}(\mathrm{state})$	10	5094.33	4112.80	308.73	‡
37	$\pi(\cdot)$, $\varphi(\mathrm{state})$, $\psi^{\mathrm{SA}} + \psi^{\mathrm{R}} + \psi^{\mathrm{B,NB}}$, $\mathrm{p}(\mathrm{state})$	10	5098.02	4115.72	311.65	‡
38	$\pi(\cdot)$, $\varphi(\mathrm{A} + \mathrm{A}^2)$, $\psi^{\mathrm{SA}} + \psi^{\mathrm{R}} + \psi^{\mathrm{B,NB}}$, $\mathrm{p}(\mathrm{state})$	11	5089.00	4116.70	312.63	‡
39	$\pi(\cdot)$, $\varphi(\mathrm{a})$, $\psi^{\mathrm{SA}} + [\psi^{\mathrm{R}} \cdot \mathrm{A}] + [\psi^{\mathrm{B,NB}} \cdot \mathrm{A}]$, $\mathrm{p}(\mathrm{state})$	36	4845.60	4127.08	323.00	‡
40	$\pi(\cdot)$, $\varphi(\mathrm{A} \cdot \mathrm{state})$, $\psi^{\mathrm{SA}} + \psi^{\mathrm{R}} + \psi^{\mathrm{B,NB}}$, $\mathrm{p}(\mathrm{state})$	12	5092.51	4127.60	323.53	‡
41	$\pi(\cdot)$, $\varphi(\mathrm{A} + \mathrm{A}^2 \cdot \mathrm{state})$, $\psi^{\mathrm{SA}} + \psi^{\mathrm{R}} + \psi^{\mathrm{B,NB}}$, $\mathrm{p}(\mathrm{state})$	14	5084.36	4137.39	333.31	‡
42	$\pi(\cdot)$, $\varphi(\mathrm{A})$, $\psi^{\mathrm{SA}} + [\psi^{\mathrm{R}} \cdot \mathrm{A} + \mathrm{A}^2] + [\psi^{\mathrm{B,NB}} \cdot \mathrm{A}]$, $\mathrm{p}(\cdot)$	11	5204.17	4207.84	403.77	‡
43	$\pi(\cdot)$, $\varphi(\mathrm{A})$, $\psi^{\mathrm{SA}} + [\psi^{\mathrm{R}} \cdot \mathrm{A} + \mathrm{A}^2] + [\psi^{\mathrm{B,NB}} \cdot \mathrm{a}]$, $\mathrm{p}(\mathrm{state})$	63	4719.13	4246.23	442.15	‡
44	$\pi(\cdot)$, $\varphi(\mathrm{A} + \mathrm{A}^2)$, $\psi^{\mathrm{SA}} + [\psi^{\mathrm{R}} \cdot \mathrm{A} + \mathrm{A}^2] + [\psi^{\mathrm{B,NB}} \cdot \mathrm{a}]$, $\mathrm{p}(\mathrm{state})$	64	4713.76	4250.10	446.03	‡
45	$\pi(\cdot)$, $\varphi(\mathrm{a})$, $\psi^{\mathrm{SA}} + \psi^{\mathrm{R}} + \psi^{\mathrm{B,NB}}$, $\mathrm{p}(\mathrm{state})$	33	5040.41	4256.89	452.81	‡
46	$\pi(\cdot)$, $\varphi(\mathrm{A} + \mathrm{A}^2)$, $\psi^{\mathrm{SA}} + [\psi^{\mathrm{R}} \cdot \mathrm{a}] + [\psi^{\mathrm{B,NB}} \cdot \mathrm{a}]$, $\mathrm{p}(\mathrm{state})$	77	4646.16	4302.16	498.09	‡
47	$\pi(\cdot)$, $\varphi(\mathrm{a})$, $\psi^{\mathrm{SA}} + [\psi^{\mathrm{R}} \cdot \mathrm{A} + \mathrm{A}^2] + [\psi^{\mathrm{B,NB}} \cdot \mathrm{a}]$, $\mathrm{p}(\mathrm{state})$	86	4665.30	4390.39	586.31	‡
48	$\pi(\cdot)$, $\varphi(\mathrm{a})$, $\psi^{\mathrm{SA}} + [\psi^{\mathrm{R}} \cdot \mathrm{a}] + [\psi^{\mathrm{B,NB}} \cdot \mathrm{a}]$, $\mathrm{p}(\mathrm{state})$	99	4597.71	4442.46	638.38	‡
49	$\pi(\cdot)$, $\varphi(\mathrm{a})$, $\psi^{\mathrm{SA}} + [\psi^{\mathrm{R}} \cdot \mathrm{A}] + [\psi^{\mathrm{B,NB}} \cdot \mathrm{a}]$, $\mathrm{p}(\mathrm{state})$	85	4765.19	4461.32	657.25	‡

Notes: Survival φ and state-transition ψ models were allowed to vary by age, or age and state (age $\cdot$ state), or remain constant $(\cdot)$. Encounter probability p was assumed to be a constant, or depend on state, time in years t, or constrained time $\mathrm{t_c}$, or time and state. ψ^{R} is recruitment probability and $\psi^{\mathrm{B,NB}}$ is the transition probability between breeding and nonbreeding states. # represents model number (also M in the main text). Age was allowed to be a factor (a) or a continuous variable that is either approximated via a linear (A) or quadratic $(\mathrm{A} + \mathrm{A}^2)$ function. QSIC represents the quasi-Schwarz information criterion, where the quasi-deviance $= \frac{\mathrm{deviance}}{\hat{c}}$ and $\hat{c} = 1.2636$. ΔQSIC is the difference between the best model (lowest QSIC) and a competing model. ΔQSIC >7 units indicated strong support for one model over another. H represents individual heterogenity that was accounted for using a mixture model and Dev stands for the model deviance.

‡ Value less than 1e-10;

[a]Evidence ratio is defined as $\exp(-(\frac{1}{2} \cdot \Delta\mathrm{QSIC})$

Table S2: Time-to-event Model comparisons based on log-likelihood and SIC

Model formula	ℓ	SIC	$\Delta\ell$	ΔSIC
$\sim$ Age + lagged$_{\mathrm{BSEA}}$ + `cluster`(ID)	-3563.94	7140.87	78.09	0.00
$\sim$ Age + lagged$_{\mathrm{BSEA}}$ + `frailty`(ID)	-3563.94	7140.87	78.09	0.00
$\sim$ Age + lagged$_{\mathrm{cCF}}$ + `cluster`(ID)	-3587.78	7188.56	54.25	47.69
$\sim$ Age + lagged$_{\mathrm{cEggs}}$ + `cluster`(ID)	-3597.63	7208.26	44.40	67.39
$\sim$ lagged$_{\mathrm{BSEA}}$ + `cluster`(ID)	-3617.14	7240.78	24.89	99.91
$\sim$ lagged$_{\mathrm{cCF}}$ + `cluster`(ID)	-3621.92	7250.33	20.11	109.46
$\sim$ lagged$_{\mathrm{cEggs}}$ + `cluster`(ID)	-3623.53	7253.56	18.50	112.69
$\sim$ AFR, `cluster`(ID)	-3635.50	7277.50	6.53	136.63
$\sim$ lagged$_{\mathrm{AEP}}$ + `cluster`(ID)	-3638.16	7282.81	3.88	141.94
$\sim$ Age + AFR + `cluster`(ID)	-3635.33	7283.64	6.71	142.78
$\sim$ 1	-3642.03	7284.06	0.00	143.20
$\sim$ lagged$_{\mathrm{CF}}$ + `cluster`(ID)	-3639.40	7285.29	2.63	144.42
$\sim$ Age + lagged$_{\mathrm{AEP}}$ + `cluster`(ID)	-3637.49	7287.98	4.54	147.11
$\sim$ Age + `cluster`(ID)	-3641.94	7290.37	0.09	149.50
$\sim$ Age + lagged$_{\mathrm{CF}}$ + `cluster`(ID)	-3638.98	7290.96	3.05	150.09
$\sim$ Age + lagged$_{\mathrm{cCF}}$ + `frailty`(ID)	-3544.29	7318.53	97.74	177.66
$\sim$ Age + lagged$_{\mathrm{cEggs}}$ + `frailty`(ID)	-3539.53	7357.39	102.50	216.52
$\sim$ Age + AFR + `frailty`(ID)	-3534.98	7470.16	107.05	329.29
$\sim$ Age + lagged$_{\mathrm{AEP}}$ + `frailty`(ID)	-3534.20	7482.77	107.84	341.90
$\sim$ Age + lagged$_{\mathrm{CF}}$ + `frailty`(ID)	-3532.85	7488.11	109.18	347.24

Model comparison for time-to-event analysis. Statistics presented include log-likelihood ℓ, Schwarz Information Criterion (SIC), and differences in ℓ and SIC relative to the best model – lowest SIC and largest ℓ. Models incorporated parameters for breeding experience (lagged$_{\mathrm{BSEA}}$), reproductive effort in terms of the number of clutches (lagged$_{\mathrm{cCF}}$) or number of eggs laid (lagged$_{\mathrm{cEggs}}$) a female has laid so far in her life. We also considered a Markov effect, whereby the time to nest again only depends on the number of clutches (lagged$_{\mathrm{CF}}$) or eggs (lagged$_{\mathrm{AEP}}$) a female laid during her last reproductive season. CF = clutch frequency, AEP = annual egg production, BSEA = breeding season number, cEggs = cumulative eggs, cCF = cumulative clutch frequency, AFR = age at first reproduction (a proxy for sexual maturity). ID is a factor for individual identification, whereas `frailty` and `cluster` account for repeated individual measurements.

4 Supplementary figures

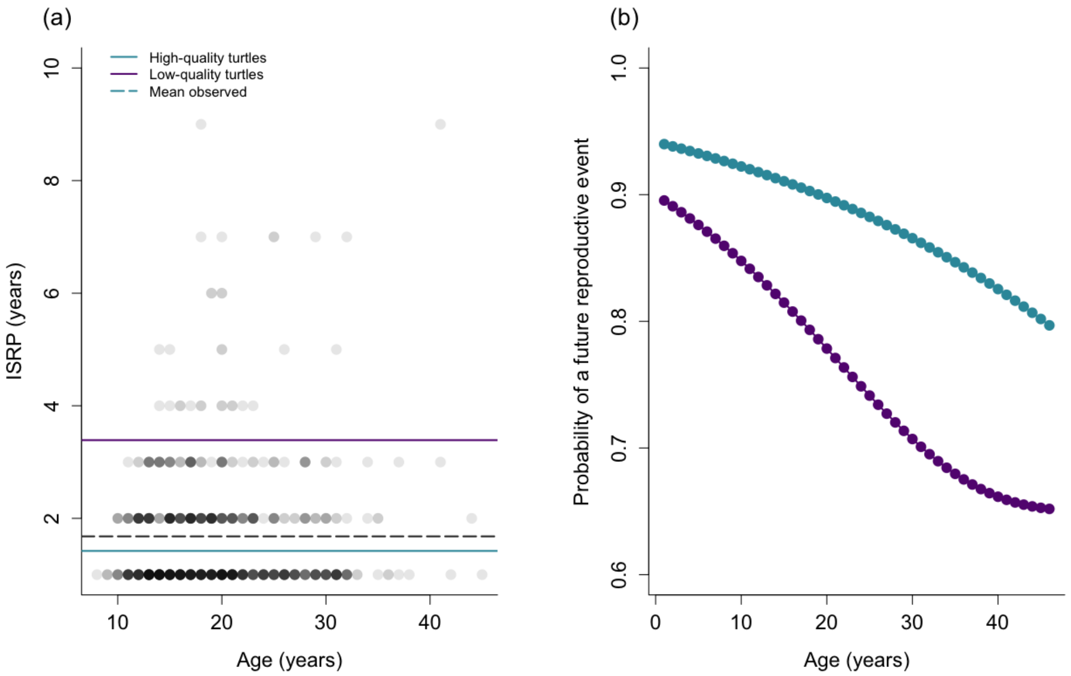

Fig. S1: (a) Changes in the inter-seasonal reproductive period (ISRP) with age for high- and low-quality turtles (classified from the MECMR mixture model). (b) Probability of a future reproductive event for adult females of differing quality.

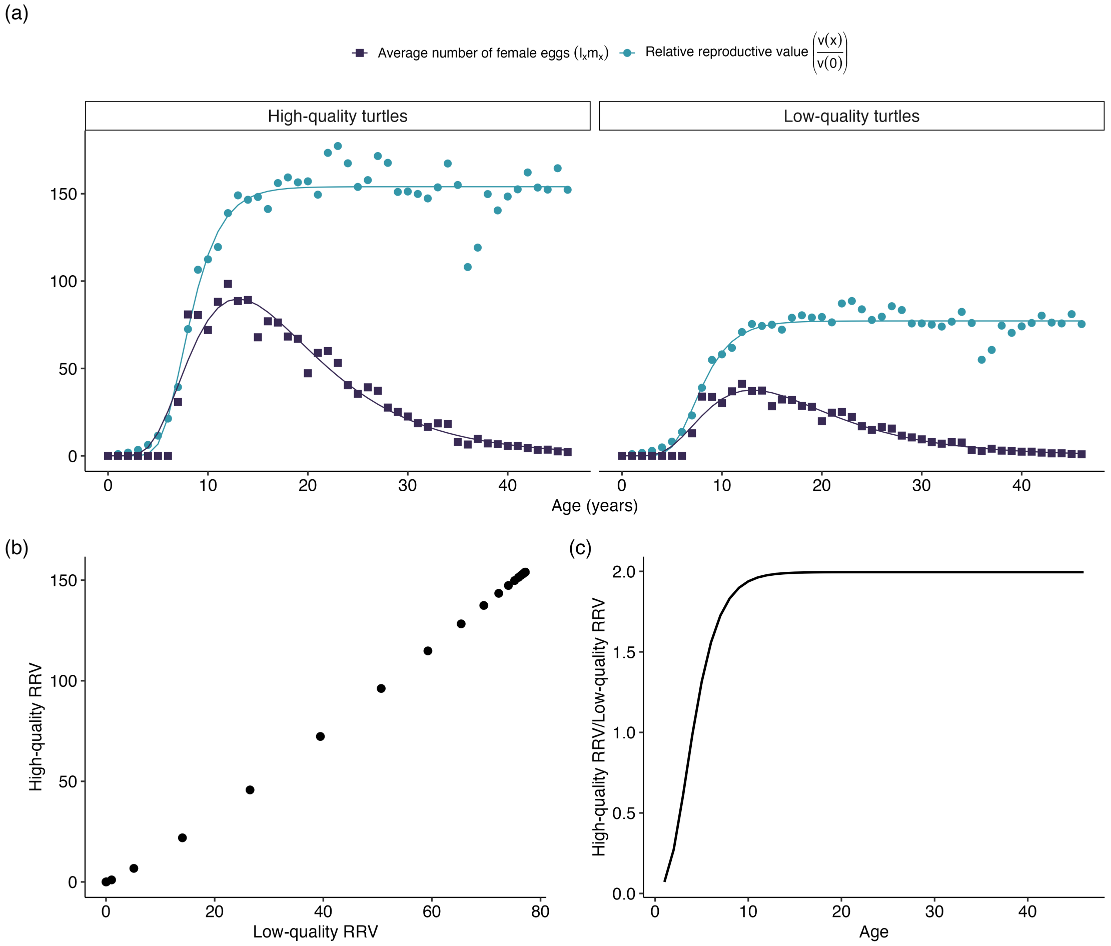

Fig. S2: (**a**) Estimates of relative reproductive value (RRV) and fecundity adjusted for survival ($l_x m_x$) by individual quality. Annual fecundity was calculated by dividing age-specific fecundity by the inter-seasonal reproductive period (ISRP) for low-quality (ISRP=3.39) and high-quality turtles (ISRP=1.42), assuming the number of eggs per breeding event was similar between groups (see main text and S1). (**b**) Comparison of RRV between the two quality groups. (**c**) The ratio of high- to low-quality RRV. In this panel, a ratio of one indicates that both groups have the same reproductive value. However, the observed ratio of approximately two after age 10 means that high-quality turtles have twice the relative reproductive value of their low-quality counterparts.
